# Supplementary material for: Estrogen‐dependent control and cell‐to‐cell variability of transcriptional bursting
Source: Mol Syst Biol. 2018 Feb 23;14(2):e7678. doi: 10.15252/msb.20177678 (PMC5825209; doi:10.15252/msb.20177678)
Supplement: Supplementary file 1 — Appendix [file MSB-14-e7678-s001.pdf]

Appendix

Estrogen-dependent control and cell-to-cell variability of transcriptional bursting

Christoph Fritzsch[1,†], Stephan Baumgärtner[1,†], Monika Kuban[1], Daria Steinshorn[1], George Reid[1,2,‡] and Stefan Legewie[1,‡,*]

[1] Institute of Molecular Biology, Ackermannweg 4, 55128 Mainz, Germany.

[2] European Molecular Biology Laboratory, Meyerhofstraße 1, 69117, Heidelberg, Germany.

[†] Co-first authors

[‡] Co-senior authors

* Lead contact

Table of contents

Appendix Supplementary Methods 2
Appendix Figures 19
Appendix Tables 30
Appendix Movies 34
Appendix Datasets 35
Appendix References 36

Appendix Supplementary Methods

Experimental methods

DNA constructs

pX330-GREB1-ex2 and pX330-GREB1-int2 (guide RNA and Cas9 expression)

Specific sgRNA sequences were designed using the CRISPR design tool (Feng Zhang, http://crispr.mit.edu), ordered as complementary oligonucleotides (gRNA_ex2_t and gRNA_ex2_b for exon 2, and gRNA_int2_t and gRNA_int2_b for intron 2), annealed and inserted in between the *Bbs*I sites of pX330-U6-Chimeric_BB-CBh-hSpCas9 (gift from Feng Zhang, Addgene plasmid #42230) to yield pX330-GREB1-ex2 for exon 2 and pX330-GREB1-int2 for intron 2 of *GREB1*.

pSB-Ubc-NLS-HA-tdPCP-tdGFP

eGFP was amplified from pHAGE-UbC-NLS-HA-tdPCP-GFP (gift from Robert Singer, Addgene plasmid #40650) using primers inF_PCPlinker_GPF_for and inF_GFP_rev. The tdPCP-GFP ORF was amplified from the same plasmid with primers inF_K2L_PCP_for and inF_GFP_PCP_rev. pmKate2-C (Evrogen, Cat#FP181) was linearized with *Xma*I and *Kpn*I and used together with both PCR products in an In-Fusion reaction (Clontech, Cat#638909) to yield the intermediate pmKate2-C-tdPCP-tdGFP. PCR with primers tdPCP_for and tdGFPSV40_rev was performed on this vector to amplify tdPCP-tdGFP with an SV40-polyadenylation sequence. The PCR product was digested with *Age*I and *Cla*I and replaced the *Age*I-*Cla*I fragment from pHAGE-UbC-NLS-HA-tdPCP-GFP yielding pHAGE-UbC-NLS-HA-tdPCP-tdGFP. The complete expression cassette was cut with *Spe*I and *Cla*I and ligated with the *Cla*I-*Spe*I digested product that resulted from a PCR on pSB-ET-iE (gift from Manfred Gessler) with the primers pSB_for and pSB_rev.

pHR-GREB1 plasmids (templates for homologous recombination)

Homology arms up- and downstream of the Cas9 cleavage site in intron 2 of *GREB1* were amplified from genomic DNA of MCF-7 cells using primers inF_int2_L_PP7_rev and inF_int2_L_pUC_for, as well as inF_int2_R_PP7_for and inF_int2_R_pUC_rev. pUC19 (New England Biolabs, Cat#N3041S) was linearized with *Hin*dIII and the 24xPP7 cassette was cut from pCR4-24xPP7SL (gift from Robert Singer, Addgene plasmid #31864) using *Spe*I and *Not*I. All four fragments were assembled using In-Fusion cloning (Clontech) to yield pHR-GREB1-int2-24xPP7. This vector was used to amplify the homology arms and the vector backbone by PCR with primers inF_loxP_R_for and inF_loxP_L_rev. The selection cassette (CMV promoter driving

expression of a bicistronic mRNA—eBFP2 carrying a peroxisomal targeting sequence, IRES, Puromycin resistance—flanked by loxP sites) is derived by two PCRs. First, IRES-Puro and the bGH polyadenylation signal were amplified from pGLUE (gift from Randall Moon, Addgene plasmid #15100) with primers inF_BFP_IRESPuro_for and inF_loxP_Puro_rev. Second, the CMV-eBFP2 cassette was amplified from pEBFP2-Nuc gift from Robert Campbell, Addgene plasmid #14893) with primers inF_loxP_CMVBFP_for and inF_IRES_BFP_rev. All three PCR fragments were assembled using In-Fusion cloning (Clontech) to yield pHR-GREB1-int2-LPIBCL. The 24xPP7 cassette was cut from pCR4-24xPP7SL using *Spe*I and *Not*I and inserted into the *Spe*I and *Not*I sites to yield the final pHR-GREB1-int2-24xPP7-LPIBCL.

To exchange the homology arms of intron 2 of *GREB1* against sequences from exon 2, the respective homology arms were amplified from genomic DNA of MCF-7 cells using primers inF_ex2_L_pUC_for and inF_ex2_L_loxP_rev for the 5' homology arm of exon 2, and inF_ex2_R_loxP_for and inF_ex2_R_pUC_rev for the 3' homology arm of exon 2. Both PCR products were used together with a PCR product with primers inF_BFP_Puro_for and inF_BFP_Puro_rev on pHR-GREB1-int2-24xPP7-LPIBCL and an *Eco*RI + *Bam*HI linearized pUC19 plasmid in an In-Fusion reaction (Clontech). The 24xPP7 cassette was cut from pCR4-24xPP7SL using *Spe*I and *Not*I and inserted into the *Spe*I and *Not*I sites of the assembled vector to yield pHR-GREB1-ex2-24xPP7-LPIBCL.

pUC-qRT-GAPDH-GREB1ex2-wt-PP7

cDNA of E2 treated MCF7-GREB1-PP7 cells was used as template for PCRs with primers inF_GAPDH_pUC_for and inF_GAPDH_GREB1wt_rev, inF_GREB1wt_GAPDH_for and inF_GREB1wt_ki_rev, and inF_GREB1ki_wt_for and inF_GREB1ki_pUC_rev. All three PCR fragments were simultaneously inserted into an *EcoR*I + *BamH*I linearized pUC19 backbone using an In-Fusion reaction (Clontech) to yield pUC-qRT-GAPDH-GREB1ex2-wt-PP7.

Image analysis

High-throughput image analysis

Images from the high content screening microscope were analyzed using the Harmony® High Content Imaging and Analysis Software (PerkinElmer, version 4.1). First, maximum intensity projections were calculated for each channel and a combined DAPI-EGFP image was calculated by summing the EGFP channel and 1/10 of the intensity of the DAPI channel (for DRAQ5: EGFP + 1/4 × DRAQ5). This image was used in the "find nuclei" building block to identify nuclei using method C (parameters: common threshold 0.45 (for DRAQ5 0.7), area 30 µm, split factor 20.5 (for DRAQ5 10.0), individual threshold 0.45, contrast 0.05). Nuclei that touch image borders

were removed and good nuclei were selected based on intensity and morphology properties (mean GFP intensity < 300, nucleus roundness > 0.65, nucleus area < 300 µm², nucleus ratio width to length > 0.45, nucleus area > 80 µm). The plane from which the maximum pixel intensity originated during the maximum intensity projection was used to discard out-of-focus nuclei. Nuclei were removed if the mean plane map value was below 4 or above 18. Spots were identified in a region that encompasses the nucleus plus a rim of 1 µm on a background subtracted image (EGFP channel - 5 px Gaussian filtered EGFP channel) using method C (parameters: radius ≤ 1.61 µm, contrast > 0.82 µm, uncorrected spot to region intensity > 2.5, distance ≥ 3 µm, spot peak radius 0.24 µm). A linear classifier was used to discriminate real transcription sites from spurious spot detections (discarded if 14.4 × spot contrast + 0.0324 × spot area + 0.126 × spot background intensity + 0.158 × spot to region intensity < 17.06). Spot and nuclei properties were exported and analyzed using custom written scripts in R. Spot intensity was calculated as the product of the corrected spot intensity and the spot area. Only the brightest spot was considered for each nucleus. A four parameter Hill-equation was fitted using the "nls" function in R with the square root of the number of cells as weights.

Live-cell image analysis and tracking of transcription sites

All live-cell image analysis was performed by custom MATLAB scripts.

Segmentation of nuclei

Nuclei were segmented based on mean intensity projections of the GFP channel. In a first step, bright nuclear foci that can adversely influence segmentation were removed by setting an intensity cut-off at the 92nd percentile on the local background-subtracted image (Gaussian smoothing with width of 30 µm). The remaining image was scaled such that the intensity values range from 0 to 1, smoothened by applying a Gaussian filter (width of 0.8 µm) and the local background was subtracted. Then, a user selected threshold (usually between 0.03 and 0.06) was applied. Holes in the resulting mask were filled and the mask was smoothened by applying an opening operation with a disk structuring element with a radius of 2.5 µm. Nuclei that were in close proximity and could not be separated by this approach were identified by size (area > 260 µm²) and a shape measure (solidity < 0.93). They were iteratively separated by identifying the best watershed lines that connect two concave regions in a nuclear mask such that they lead to a separation of clustered nuclei (described in Stoeger et al., 2015). Finally, objects smaller than 60 µm² or bigger than 1300 µm² were removed.

Tracking of nuclei

Nuclei were identified in each frame of a time-lapse movie as described above. The result of the nuclear segmentation of the previous time point was used to correct possible errors in the segmentation of the next time point. The pixel-based overlap of all individual nuclear masks of both time points were calculated and used to correct possible errors such as over- or undersegmentation of nuclei, as well as to detect disappearance or appearance of nuclei over time. After finding frame-to-frame correspondences in the corrected masks, incompletely tracked nuclei, i.e. dividing or dying cells, were discarded. Nuclei that touched image borders or that showed erroneous tracking were manually removed. In an experiment with a cell density of 80 % about 30 nuclei could be completely tracked from a typical movie.

Bandpass filtering

A two-dimensional bandpass filter was used to reduce background and pixel noise in images. Edges of the image were replicated, the image was transformed into Fourier space and multiplied with a filter that was calculated as follows:

$$BP_{x,y} = \frac{1}{1 + \left(\frac{2d_{x,y}C_{HP}}{w + h}\right)^{12}} - \frac{1}{1 + \left(\frac{2d_{x,y}C_{LP}}{w + h}\right)^{4}}$$

with w and h being the width and height of the filter, respectively, $d_{x,y}$ being the distance of the pixel at position x/y to the center of the image, and C_{HP} and C_{LP} are the cutoffs (in px) for high pass and low pass, respectively.

Spot detection

Spots were detected on maximum intensity projections of bandpass-filtered images. A bandpass of 0.6 µm to 4 µm was applied to remove high-frequency noise and low-frequency background fluorescence, e.g. of unbound nuclear tdPCP-tdGFP. Spot detection was performed using the u-track package for MATLAB in version 2.0 (Jaqaman et al., 2008) with a user-defined width of the point-spread-function (0.4 µm) and a p-value cut-off of 0.13.

Tracking of transcription sites

Transcription sites were tracked relative to the movement of the nucleus, consisting of translation and rotation, both of which were inferred from the nuclear outlines resulting from the nuclear tracking algorithm. The positions of all spots that were not more than 1 µm away from the nucleus were transformed such that they were fixed relative to a given nucleus. Then, tracklets were generated using the u-track package for MATLAB using a linear motion Kalman filter with a search radius of 6 without merging or splitting and without gap closing. Only resulting

tracklets that cover at least four time points were considered in the tracklet linking step. The cost for all possible combinations of linkages of the up to 18 longest tracklets was calculated as:

$$cost = \sum_{t=1}^{260} \frac{10 i_t}{i_{max}} + n_{gaps} + \sum_{alllinkages} \frac{2d^2}{\Delta_t}$$

with i_t being the u-track spot intensity in frame t, i_{max} being the 90 % percentile of spot intensities in the same nucleus, n_{gaps} being the total number of frames without tracklet, d being the distance between end and start of consecutive tracklets (in px), and Δ_t being the number of frames in between consecutive tracklets. The cost was set to infinite if any two tracklets overlap in time. The combination of tracklets with the lowest cost was chosen, remaining tracklets were added if this lowers the cost further, the gaps in between tracklets were interpolated linearly, and the positions were retransformed into the original coordinates of the movie. A position for background estimation was set to the centroid of the nuclear mask and shifted such that it is at least 3 µm away from the position of the transcription site.

All generated tracks were reviewed and erroneous assigned positions were corrected manually. Cells where the transcription site moves out of focus or divides (S/G2 phase of cell cycle) during acquisition were discarded.

Quantification of fluorescence intensities

Fluorescence intensities were quantified on bandpass filtered images (0.4 – 4 µm) to reduce pixel noise and background of unbound tdPCP-tdGFP. Spot intensities were quantified by fitting the width (σ_{xy} and σ_z separately) and amplitude of a 3-dimensional Gaussian distribution with an offset to the intensity in the image stack in a circular window with a diameter of 1.9 µm centered around the tracked position. The squared error was minimized using the “fminsearchbnd” function (John D'Errico, https://www.mathworks.com/matlabcentral/fileexchange/8277-fminsearchbnd--fminsearchcon, February 2014) with σ_{xy} being constrained to 0.1 – 0.4 µm and σ_z to 0.3 – 1 µm. The integrated intensity was calculated from the fitted parameters.

Extraction of morphological features

Cellular area was calculated from hand-drawn masks in the brightfield channel at the beginning of each movie. Morphological features of nuclei were calculated for every 10th frame of the movie as follows. Nuclei were identified as described above and area, eccentricity, and solidity were calculated using the “regionprops” function in MATLAB. Cell density was calculated as the number of nuclei that are within a circle of 200 or 400 pixels around the centroid of each nucleus and normalized to the actual area of the circle that is visible in the image. Mean nuclear GFP

levels were obtained as the mean intensity of all pixels of a maximum intensity projection that fall within the nuclear mask. Multilinear regression was performed using the “lm” function in R.

Single-molecule RNA FISH image analysis

All image analysis was performed by custom MATLAB scripts.

Segmentation of nuclei

Nuclei were detected on maximum intensity projections of images in the DAPI channel. Intensity inhomogeneity was removed by local background (Gaussian smoothing, width of 10 µm) subtraction. After scaling of intensities to range from 0 to 1, global thresholding using Otsu’s method (Otsu, 1979) was applied. Holes in the mask were filled and the mask was smoothened by applying an opening operation with a disk structuring element with a radius of 2.5 µm. Clustered nuclei were identified (area > 175 µm², solidity < 0.935) and separated as described above. Nuclei that were smaller than 80 µm² or bigger than 250 µm² were removed and the remaining objects were inflated by 4 px. Nuclei with a mean DAPI intensity of more than 3000 and a solidity of less than 0.96 were not considered for further analysis.

Spot detection and quantification

Spots were detected on maximum intensity projections of bandpass-filtered (0.4 µm to 4 µm) images. Spot detection was performed using the u-track package for MATLAB in version 2.0 (Jaqaman et al., 2008) with a point-spread-function width of 0.12 µm and an alpha-value of 0.1. Spots with an amplitude above 0.004 (Quasar® 570 and GFP) or 0.0015 (Quasar® 670) were quantified by fitting a three-dimensional Gaussian function to the 3D image stack within a circular window of 1.4 µm with σ_{xy} being constrained to 0.08 – 0.38 µm and σ_z to 0.27 – 1.35 µm. Spurious spots (σ_{xy} < 0.12 µm, amplitude < 300 (Quasar® 570) or < 100 (Quasar® 670 and GFP), ratio of amplitude and (σ_{xy}–1) < 800 (Quasar® 570), < 200 (Quasar® 670), or < 150 (GFP) were discarded.

Absolute quantification by calibration to intensities of single RNAs

In smRNA FISH images for *GREB1* exons single transcripts are visible as diffraction-limited spots in the cytoplasm. The intensity of these spots was used to calibrate the intensities of transcription sites and derive absolute nascent RNA numbers. A lognormal distribution was fitted using the “lsqnonlin” function in MATLAB to the intensities of spots that are located outside of nuclei and its mean was used as the intensity of a single RNA

Computational methods

All modelling and data analysis was implemented in the python programming language (Van Rossum and Drake, 2003) in combination with the IPython package (Perez and Granger, 2007). Furthermore the NumPy (van der Walt et al., 2011), SciPy (Jones et al., 2001) and Matplotlib (Hunter et al., 2007) libraries for scientific computation and visualization were used. The python code and IPython notebooks to run simulations and model fits can be found on GitHub (https://github.com/baumgast/gene_transcription_SMC_ABC).

Hybrid model to understand stimulus dependent single cell transcription

We compared promoter cycles of different complexity in their ability to explain experimental data. Fig 3A shows all model topologies, with the smallest possible cycle of only two states being shown on the left, whereas longer cycles are shown representing models of higher complexity. The three-state model has two inactive promoter states and the four-state and one one of the ten-state models incorporates an additional ON-state. By this scheme, the set of model topologies can be extended by adding more states in either the active or inactive phase of the cycle. Switching between states occurs stochastically with the rates k_{ON} and k_{OFF}, respectively, and is an irreversible ratchet-like process. We used the well-known stochastic simulation algorithm (Gillespie, 1977) to run forward simulations of the different models.

Due to the structure of the artificial transcript, the fluorescent signal visible under the microscope is delayed relative to transcriptional initiation. RNA polymerase first has to transcribe the stem-loop section of the reporter construct for the fluorescent coat protein to bind and visualize it (see Fig 1B). This generates a first delay τ_1. After a second delay τ_2 RNA polymerase has completed the transcript which then falls off the transcription site and is not contributing to the detected signal any more.

To gain computational speed, we used a hybrid stochastic-deterministic method. Instead of stochastically simulating the mRNA elongation by multiple extra states, we used deterministic elongation. The deterministic signal that is created by a single transcript is completely defined by the structure of the transcript (length, position of the stem-loops) and the velocity of the RNA polymerase as depicted in Figs. 1B and 3A. RNA polymerase velocity is assumed constant over the whole transcript. Thus, the position of the stem-loops within the transcript defines the first delay τ_1 between transcript initiation and appearance of the fluorescence signal. The length of the transcript defines the second delay τ_2 when the transcript is finished and released from the transcription site. The stochastic part of the simulation created the time points of transcriptional initiation events and the final time course consisted of the sum over all initiated single transcript

signals. Hybrid stochastic-deterministic simulations showed only minor deviations from fully stochastic simulations and yielded an approximate 50-fold acceleration in computation time (not shown).

Intensity of single transcripts relates RNA simulations with experimental data

Stochastic simulations of the model produce RNA counts over time whereas the experimental readout is fluorescence light intensity. To compare both, we measured the intensity of a single transcript and used this as a factor of proportionality α between simulated RNA count and measured light intensity.

As a measure of background light intensity, a site within the cell nucleus distant from the transcription site was quantified in each image. The distribution of background light intensities closely follows a lognormal distribution with similar shape and scale parameters under all experimental conditions. With the so calibrated noise model, the probability to observe signal *S* given *R* transcripts at the transcription site is given by:

$$P(S|R) = \alpha R + noise$$

$$noise \sim Lognormal(scale, shape)$$

where the noise is sampled from a lognormal distribution with the *scale* and *shape* parameter obtained by fitting to the background noise distribution.

Model parametrization

Model parameterization closely follows the approach of Zoller et al. (2015). The transcriptional model in Fig 3A is parameterized by the number of active states *N*, the number of inactive states *M*, the index of the extrinsic noise source *e,* the initiation rate k_{init}, the transition rates k_{ON} of the active states and the transition rates k_{OFF} of the inactive states. The first three parameters describe the model topology and the remaining parameters the kinetics.

The parametrization of the kinetic part can be expressed by the parameters b, t_{ON}, t_{OFF}, π_i, p_j. Where $b = k_{init} \cdot t_{ON}$ is the burst size, t_{ON} and t_{OFF} are the total ON- and OFF-times respectively. $\pi_i = t_{ON_i}/t_{ON}$ and $p_j = t_{OFF_j}/t_{OFF}$ are the fractions of time spent in the *i*-th active or *j*-th inactive state. Therefore, the total ON- and OFF-times are given by: $t_{ON} = \sum t_{ON_i} = \sum \frac{1}{k_{ON_i}}$ and $t_{OFF} = \sum t_{OFF_j} = \sum \frac{1}{k_{OFF_j}}$. The fractions π_i and p_j follow the constraint $\sum p_j = \sum \pi_i = 1$.

To take the symmetry of the promoter cycle into account, an ordering condition on the π_i and the p_i is assumed: $\pi_1 \geq \pi_2 \geq \cdots \geq \pi_N; p_1 \geq p_2 \geq \cdots \geq p_M$. Such an ordering can be achieved by the following parametrization:

$$p_2 = u_1 p_1$$

$$p_3 = u_2 p_2 = u_1 u_2 p_1$$

$$\cdots$$

$$p_M = u_{M-1} p_{M-1} = p_1 \prod_{j=1}^{M-1} u_j$$

where $u_j \in [0, 1]$. Together with the constraint $\sum p_j = 1$ the number of free parameters u_j and p_j is the same and the latter can be expressed by the former. With the system of equations:

$$p_M = 1 - \sum_{j=1}^{M-1} p_j = 1 - p_1 \left(1 + \sum_{l=1}^{M-2} \prod_{j=1}^{l} u_j \right)$$

$$p_M = p_1 \sum_{j}^{M-1} u_j$$

p_1 can be expressed as:

$$p_1 = \frac{1}{1 + \sum_{l=1}^{M-1} \prod_{j=1}^{l} u_j}$$

All other p_j can then be calculated as:

$$p_k = \frac{\sum_{j=1}^{k} u_j}{1 + \sum_{l=1}^{M-1} \prod_{j=1}^{l} u_j}$$

An equivalent parametrization was used for the π_i of the active transcription phase.

Implementation of extrinsic noise sources

Individual cells showed a high variability in their transcriptional output at the same experimental condition as displayed in Fig 2A and EV2A. This cell-to-cell variability between cells can be introduced into the model by perturbations of individual model parameters. The underlying assumption is that the cellular state among different cells varies even under the same

experimental conditions. While simulating a cell population, one or more parameters of the model can be perturbed by resampling from a certain distribution, i.e. for each simulated cell an individual parameter would be sampled. Such perturbations represent extrinsic sources of variability like varying protein copy numbers among cells within a population. Perturbations are assumed to be stable over time, i.e. do not change over the course of the simulation, as cell-to-cell variability showed to be temporally stable (Appendix Fig S3).

We considered individual perturbations on the polymerase elongation rate, the initiation rate, the ON-time and the OFF-time. For instance, varying ATP levels could be responsible for varying polymerase elongation, varying copy numbers of RNA polymerase could cause different initiation rates. Copy number variations in other proteins necessary to activate transcription could cause variations in ON- or OFF-times. Besides single perturbations, we included combinations of two of these perturbations.

Perturbed RNA polymerase II elongation rates were sampled from a uniform distribution within the range of velocities reported in the literature, i.e., $k_{elong} \sim Unif(1{,}5)$ kb/min. All other parameters were perturbed by sampling from normal distributions. The standard deviation $\sigma_{(k_{init}, t_{ON}, t_{OFF})}$ of that normal distribution is a measure for the strength of the perturbation and is an extra parameter of the model. To avoid negative values the absolute value of the sampled parameter was taken.

Approximate Bayesian Computation for likelihood-free model calibration

Calibrating the model by fitting to experimental data consists of two tasks: model selection and parameter estimation. The former will reveal the most likely model topology and extrinsic noise source, the latter the corresponding parameter values. This section will introduce a likelihood-free Bayesian method to calibrate stochastic models to data. In addition to parameter estimation, this method easily allows to incorporate model selection.

Calibration of stochastic models is not straight forward and complicated by the difficulty to calculate for a given dataset D the likelihood $L(\theta, D)$ of the parameter vector θ. Approximate Bayesian Computation (ABC) provides a way to circumvent the calculation of a likelihood by comparing simulations with experimental data by a distance measure $\rho(D_{sim},D_{exp})$ and thus returns an approximate posterior distribution (Beaumont et al., 2002; Tavaré et al., 1997). In contrast to maximum likelihood model fitting, a Bayesian approach provides not only a point estimation of model parameters together with a confidence interval but yields a full posterior distribution of acceptable parameter estimations. In addition, ABC allows to treat the model as

an extra parameter and thus, to include model selection into the actual fitting yielding a posterior distribution on the model as well. The simplest ABC algorithm is a rejection algorithm where model *m* and a suitable parameter set θ are sampled from the prior distribution and a synthetic dataset is simulated. If the distance measure ρ is below a predefined threshold ε, the pair (m,θ) is accepted. By repeated sampling, simulation, and comparison, it is possible to gain a combined posterior distribution $P\ (m,\theta|\rho(D_{sim},D_{exp})\ \leq\ \varepsilon)$ on the model and the corresponding parameters. For a sufficiently good approximation of the posterior, a small distance threshold ε is required, making it necessary to simulate an enormous number of candidate parameter sets. A too high threshold would result in a posterior distribution that resembles the prior without revealing new insight. The actual numerical value of ε depends on the type of distance measure.

Features of experimental data used for model calibration

Global distribution of intensity values

The global intensity distribution shows a characteristic shape as shown in Fig 2D. This feature ignores the time course nature of the data and thus resembles a univariate distribution that can easily be compared to a simulated distribution utilizing the Kolmogorov-Smirnov statistic. This statistic is the maximal vertical distance between the cumulative distribution functions of the distributions to be compared, i.e. it is a number between zero and one, with zero representing perfect agreement.

Mean autocorrelation functions

In contrast to the global histogram, the autocorrelation function (ACF) incorporates the time course nature of the measured signal. It describes the similarity between measured intensities as a function of the time lag between them. The mean ACF over all cells characterizes the population average of this similarity. The ACF was calculated according to the usual definition in statistics

$$ACF(\tau) = \frac{E[(X_t - \mu)(X_{t+\tau} - \mu)]}{\sigma^2}$$

using a sliding window approach, with X_t being the intensity value at time point t, μ the mean and σ the standard deviation of the measured light intensity of a single cell. The observed time courses contained 250 data points and a window of 125 data points was slid along the time course with a step size of 5 data points resulting in 25 window positions. For each window position, the ACF for each cell was calculated and then averaged over all cells. Non-responding cells show an ACF immediately dropping to zero. Those cells cause the characteristic kink in the

mean ACF at a lag of one (Appendix Fig S4). The distance between data and simulation was calculated by the sum of squared distance between the mean ACF of data and simulations.

$$dist_{ACF} = \sum_{i=1}^{N} \left(acf_i^d - acf_i^s\right)^2$$

d and *s* denote data and simulation, respectively. *N* is the maximum lag up to which the mean ACF should be considered. Here a value of *N = 51* minutes was chosen, which corresponds to the 17th data point.

Distribution of ACF half lives

A main feature of cell-to-cell variability is the single-cell distribution of the half-life of the ACF. Half-life of the autocorrelation function here denotes the time at which the ACF drops from a value of 1 to 0.5 and can be used as a measure of the variability in temporal behavior among the cells of one dataset. Non-responding cells would all have a half-life of 1.5 min corresponding to half the imaging interval. Responding cells display a much wider variability.

For each window position of the sliding window during calculation of the autocorrelation function, the distribution of the ACF half-lives was estimated. The distance between simulation and data was calculated by the Kolmogorov-Smirnov statistic.

Distribution of ACF values at a lag of one

A second ACF-based measure of cell-to-cell variability is the distribution of the ACF values at a lag of one. Non-responding cells would show a value of zero, strongly responding cells a value close to one. Thus, this measure reflects the partitioning of the observed cells into responding and non-responding ones. The distance between simulations and data for this feature was calculated via the Kolmogorov-Smirnov statistic.

Maximum mean discrepancy

The last metric is the maximum mean discrepancy (MMD), which is a statistic to compare multivariate distributions (Gretton et al., 2012). We used a published python script to calculate MMD for the datasets and corresponding model simulations (Asch, 2012).

As total distance between simulation and experimental data, the sum of all five metrics was utilized. Smaller distance values represent better agreement between simulation and data. A distance value of zero, however, is highly unlikely due to the stochastic nature of the process, i.e. two simulations with the same parameters will not yield an identical outcome.

Distance of an optimal fit

Before running actual model fits, it was necessary to determine the distance measure of a good fit. Due to the intrinsic variability of the stochastic simulations, even two datasets created with the same model and parameters would not match exactly. To assess the characteristic self-distance, we simulated 200 datasets for each of the 22 different models that we used for algorithm benchmarking. For each dataset, we selected 500 random pairs and calculated their mutual distance (Fig EV3A). From these distributions, we estimated a value of 0.5 as a distance value for an optimal fit.

Sequential Monte Carlo Approximate Bayesian Computation

Sequential Monte Carlo Approximate Bayesian Computation (SMC ABC) provides a more efficient way to perform ABC than the simple rejection algorithm introduced above by approaching the true posterior distribution sequentially via a series of intermediate distributions (Del Moral et al., 2006; Sisson et al., 2007). The approach implemented here follows Toni et al. (2009).

Model and parameter prior distributions

As with all Bayesian approaches, a prior belief about the model topology and the corresponding parameters has to be specified. The full prior is a multivariate distribution of all parameters that is difficult to sample from. A common way to circumvent this is to factorize the full prior of the system into a product of priors of the individual parameters. In addition to the parameters, a prior on the model *m* itself has to be specified as well.

$$\pi(m, \theta_m) = \pi(m)\pi(k_m)\pi(t_{ON})\pi(t_{OFF})\pi(\mu_i|m)\pi(u_j|m)$$

For the model and the parameters μ_i and u_i that describe the fraction of time that the system spends in individual states of the ON- or OFF-phase and the strength of the parameter perturbation, respectively, we used a uniform prior. In case of the μ_i and u_i over the interval from 0.8 to 1 and for the perturbation strength over the interval from 1 to 8. The prior initiation rate was sampled from a lognormal distribution with parameters μ = 5 and σ = 0.8. We used exponential distributions as priors for the total promoter ON- and OFF-times with mean values of 10 and 70 minutes respectively.

Creation of an initial particle population from the prior

In an initial iteration, a start population of particles was sampled from the prior distributions of the model and the parameters. A particle consists of a weight, a unique model index, and the corresponding parameters. For each particle, a dataset was simulated and compared with the

experimental data. This initial round is essentially the simple rejection algorithm introduced before. To ensure a dense initial sampling of model and parameter space, a population of 50000 candidate particles was sampled from the model and parameter prior distributions. Before each SMC ABC run, the 2000 best candidate particles were selected from this initial population to gain a start population.

Creation of new particles with proposal distributions

From each iteration, the best 20 % of particles were taken to the next round and the new population was filled up to its initial size with particles being created out of the accepted particles using proposal distributions. The particle with the largest distance within the best 20 % of particles from the previous iteration defined the threshold distance ε_t of the current iteration.

Particle creation was done by proposing new particles in the proximity of accepted particles using proposal distributions. First, a particle from the accepted population was selected at random based on its weight, and subsequently, the particle parameters including the model were changed according to the proposal distributions. For each particle, a dataset was simulated and compared with the experimental data. If its distance was smaller than the current maximal distance ε_t the particle was added to the updated population. The weight of each new particle was calculated based on the particles from the previous population and the prior probability of the particle. It is a measure for the probability of the particle to reach its position based on the positions of its predecessors combined with the prior belief of possible particle positions within parameter space.

Particle creation in each iteration was carried out in several rounds. In each round, the number of created and simulated particles was determined by the number necessary to fill up the population to its initial size. No further rounds were performed if the particle population reached its initial size with all particles having a distance that is smaller than the maximal distance ε_t. To avoid extremely long computations the maximal number of rounds was limited to twenty in each iteration, as this setting proved to be useful in benchmark runs. In case the population could not be filled up with particles below the distance ε_t it was filled up with the best particles above ε_t.

If particle creation changed the model topology and the newly created particle had less promoter states after the proposed move than before, the fastest states were omitted, i.e. the corresponding μ or u_i were deleted from the parameter vector. In the opposite case, when new states had to be added, new values for μ or u_i were sampled from their prior. For the parameter describing the strength of perturbations on parameters, i.e. extrinsic noise, new values were

proposed with a normal distribution $N(\sigma', 1)$ in case when the perturbation after the model change $\sigma_{(k_{init}, t_{ON}, t_{OFF})}$ remained the same. In case the perturbation changed, a new $\sigma_{(k_{init}, t_{ON}, t_{OFF})}$ was sampled from its prior.

Proposal distributions of the kinetic parameters again closely followed the work of Zoller et al. (2015). The parameters b, t_{ON} and t_{OFF} were changed with a lognormal distribution $LN(\theta;\theta',\sigma_\theta)$ with individual scale parameters σ_θ. As proposal distribution for the u_i, a beta distribution $q_\beta(u,\alpha(u'),\beta(u'))$ was used, where the parameters of the distribution were defined as: $\alpha(u') = 1+\lambda u'$ and $\beta(u') = 1+\lambda(1-u')$ with $\lambda = 2$. A value of two proved useful in benchmark tests of the algorithm.

The algorithm terminates when no improvement is gained

By iterating this algorithm, the true posterior is approached by only accepting particles that are improving the distance measure with respect to the previous iteration. The algorithm terminates either when all particles are below a final stopping distance or when the improvement of distance between subsequent iterations is less than 5 %.

For model fitting of steady-state data the initial state of the promoter was sampled randomly and the first 80 minutes were discarded for comparison with experimental data to ensure random initial conditions. We used simulations of synchronized cells, i.e. cells that all started in the first OFF-state, to investigate transcriptional response times after estrogen induction and to fit data from induction experiments.

Algorithm benchmarking

To assess the ability of the SMC ABC approach to recover the correct model and parameter values, we turned to synthetic benchmark datasets. In total, we simulated 22 different datasets using different model topologies and various parameter values. Fig EV3A lists all datasets, with boxplots showing simulated self-distances (orange) and final distances after SMC ABC model fitting (grey). For all datasets it was possible to reach final distances close to the theoretical optimum. Fig EV3B shows the parameter posterior distributions yielded by SMC ABC on the benchmark datasets. During benchmarking, we varied all three main parameters (burst size, ON-time and OFF-time) independently. Transcription initiation rate as ratio of burst size and ON-time is shown for completeness. In general, the SMC ABC algorithm performs well in parameter estimation. We tried to assess parameter limits by simulating datasets with either very long (700 min) or short (10 min) promoter OFF-times. While an OFF-time of 700 minutes did not

impose a problem to the algorithm, it was difficult to distinguish OFF-times of 10 or 15 minutes, representing the lower limit.

Overall, the model selection of our algorithm performs well (Fig EV3C). At short OFF-times (i.e., high estrogen concentration), however, model selection shows a tendency to eliminate extrinsic noise sources, thereby choosing simpler model variants than those used for generating the synthetic data.`

Creation of global start populations out of posterior particles of individual fits

The results of fitting experimental datasets at all E2 doses separately (Fig 3) revealed a substantial overlap of the parameter posteriors (except the OFF-time). This suggested that it is possible to fit one global model to all datasets, in which all parameters except the OFF-time were assumed to be the same over all estrogen doses (Fig. 6). The advantage of such an approach is its generality: it provides a minimal model with less parameters and avoids overfitting. If the same model can explain different experimental conditions, this would provide a mechanistic understanding of transcriptional regulation in response to alterations in estrogen concentration.

The first step in fitting a global model with the SMC ABC algorithm was to generate a sufficiently good start population of particles by filtering the results of the individual fits. Here we give an overview how we created candidate starting particles from the posterior particles of model fits to individual datasets on the example with the promoter OFF-time as local parameter. We used a similar approach when testing for the initiation rate as a local parameter.

First, for each dataset, all posterior particles favoring the two-state model with k_{elong} and k_{init} as extrinsic noise source (model topology 1-1-5) were selected. Second, the resulting particles were further filtered for overlap in the posterior distributions of the transcription initiation rate and the promoter ON-time (Fig. EV3E). The overlap between posterior distributions of the initiation rate was wide and thus the filtered range was from 1 to 20 minutes^{-1}. The overlapping region for the promoter ON-time was narrow and limited to 0.3 to 1.5 minutes. Each of the filtered particles already had promising values for the global parameters initiation rate k_{init}, strength of cell to cell variability $\sigma_{k_{init}}$ and promoter ON-time t_{ON}. In contrast, the OFF-time was still undefined and had to be estimated separately for each of the eight E2 concentrations. To create a global start population, good combinations with the local parameter (promoter OFF-time) were estimated by a parameter scan. For each of the filtered particles, multiple simulations with increasing OFF-times were generated and compared with the eight datasets by calculating their summed

distance over all 5 features described above. Candidate values for the OFF-time during the parameter scan were selected based on the posterior distributions of the individual fits leading to a series of 19 values from low (1 minute) to high (1200 minutes). OFF-time combinations yielding the smallest joint distance to all data sets were added to the final particles, each OFF time corresponding to one estrogen concentration. Thus, a particle in global fitting consisted of the global and the local parameters. In this way, the most promising combinations of global and local parameters were found. During global model fitting multiple simulations were run for each particle, one for each local parameter. Each simulation was compared to its corresponding data set and the distance calculated. The global distance was then the sum of all individual distances. We used a similar approach to create a particle start population when fitting a global model with burst size as global parameter.

Calculation of extrinsic and intrinsic noise contributions

The total noise level can be estimated by the ratio of mean and standard deviation known as the coefficient of variation (CV). Extrinsic and intrinsic noise are additive by:

$$CV_{tot}^2 = CV_{int}^2 + CV_{ext}^2$$

We estimated the total noise from the different data sets directly by calculating mean and standard deviation. Before calculation of the individual noise contributions we subtracted the mean background signal from the data. The variation in the total RNA output from the whole observation time provides a measure for the extrinsic noise. Total RNA output was derived from the area under curve for every cell, which was calculated using the trapezoid function, and dividing this calculated area by the average area produced by a single transcript. The intrinsic noise is then simply the difference between total and extrinsic noise. We repeatedly calculated both total and extrinsic noise via bootstrapping.

Appendix Figures

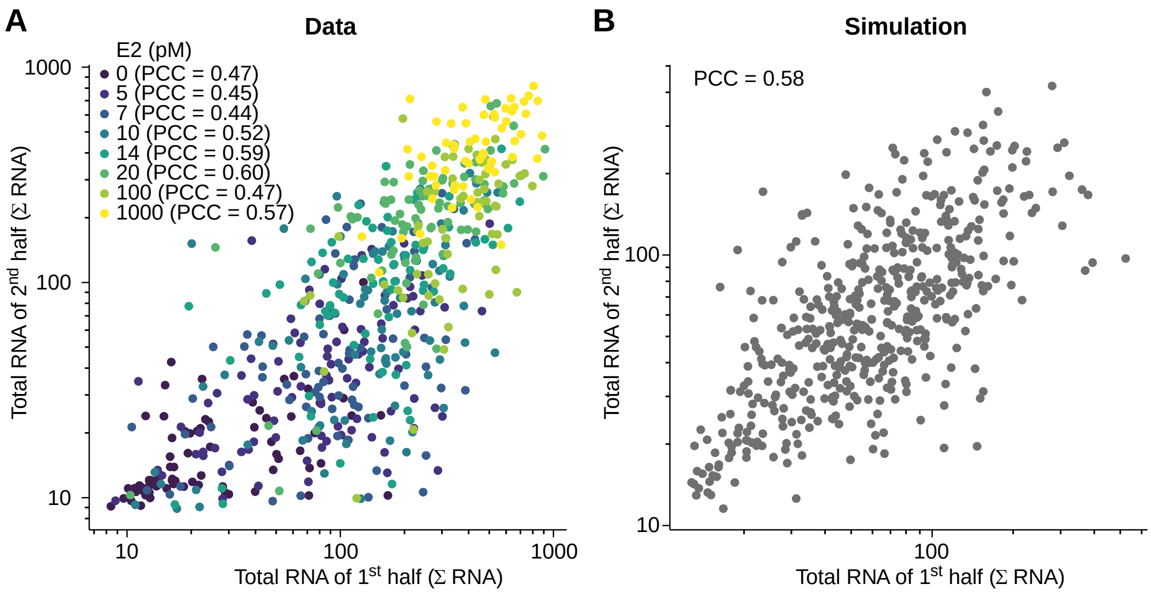

Appendix Figure S1. Correlations in total RNA output are comparable for experiments and simulations that are based on the assumption of a temporally stable extrinsic noise. Related to Fig 3.

(A) The total number of RNAs produced during the first and second half (6 h) of each single cell fluorescence trajectory was determined, and their Pearson correlation coefficient (PCC) over all cells was calculated (separately for each estrogen concentration). Positive correlations in total RNA output indicate temporal stability of extrinsic noise.

(B) Stochastic simulations were carried out (t_{ON} = *0.8 min;* t_{OFF} = *40 min; b = 8 RNAs/burst*; model topology: 1-1-5) with resampling of k_{init} and k_{elong} for each cell, and the correlation in total RNA output between first and second half was calculated as in A.

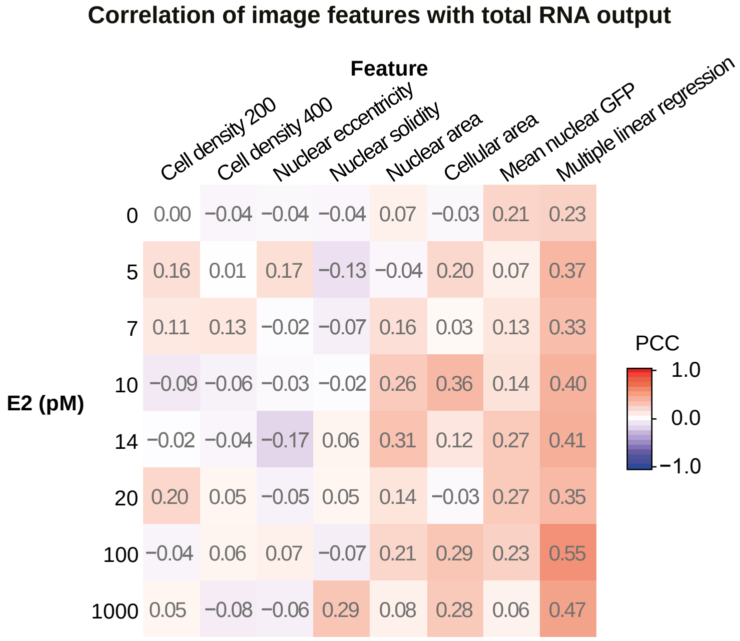

Appendix Figure S2. Morphological features account only for a small part of extrinsic variations in RNA output. Related to Fig 2.

Cellular area, morphological features of nuclei, and descriptors of cell density were used to predict the total RNA output (see Appendix Supplementary Methods for details). The Pearson correlation coefficient (PCC) was calculated for each E2 concentration and each feature separately, as well as for a multilinear regression model in which all features enter as a linear combination with the coefficients being estimated by fitting to the total RNA output over all cells. The Pearson correlation coefficient for the multilinear regression is higher (0.4-0.55 at higher E2 concentrations) than for individual features. This indicates that multiple cellular features independently control transcriptional activity, although the identity of these contributing factors varied across E2 concentrations.

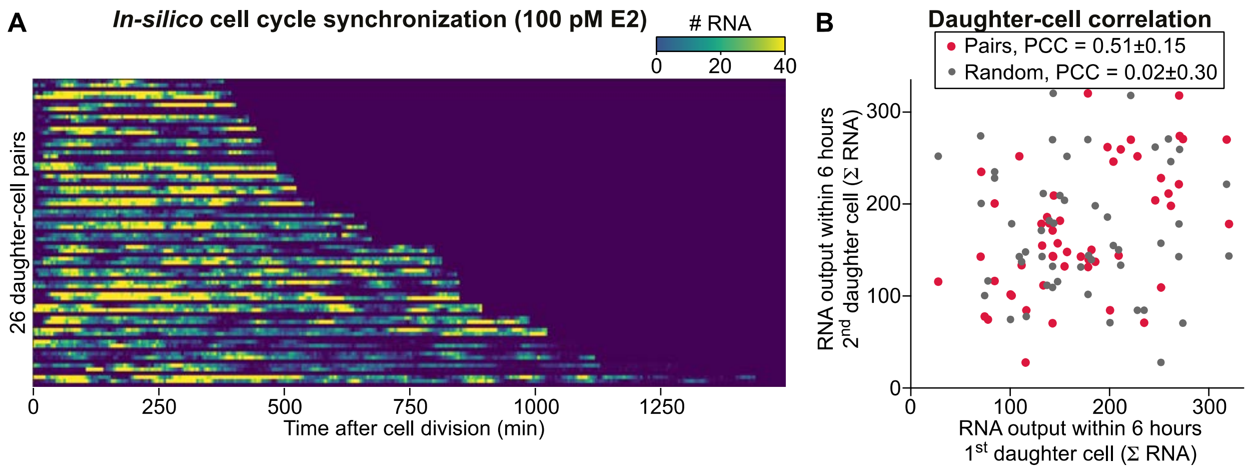

Appendix Figure S3. Extrinsic cell-to-cell variability is retained among cells in early G1 phase of the cell cycle and correlates between daughter cells. Related to Fig 2 and Fig 5.
(A) MCF7-GREB1-PP7 cells were imaged every 5 minutes for 25 hours to observe cell divisions and enable tracking of conjugate daughter cells. *GREB1* transcriptional activity was quantified as before and transcriptional trajectories of daughter cells were aligned to the time of cell division (sorted by the total observation time), with a dark line separating two daughter cell trajectories. The variability (CV^2) of total RNA output (0.16) is similar to unsynchronized cells at 100 pM E2 (0.19, see Fig 2A). In contrast, the bursting kinetics of sister cells are not correlated (not shown).

(B) Total RNA output was calculated for the first 6 hours after cell division and the Pearson correlation coefficient (PCC) was calculated for all daughter-cell pairs (red) and randomly reassigned pairs (gray), with mean ± standard deviation from bootstrapping. Correlation in total RNA output indicates inheritance of extrinsic noise, e.g. through a diffusible factor. Symmetry in the plot arises from switching of cell pairs.

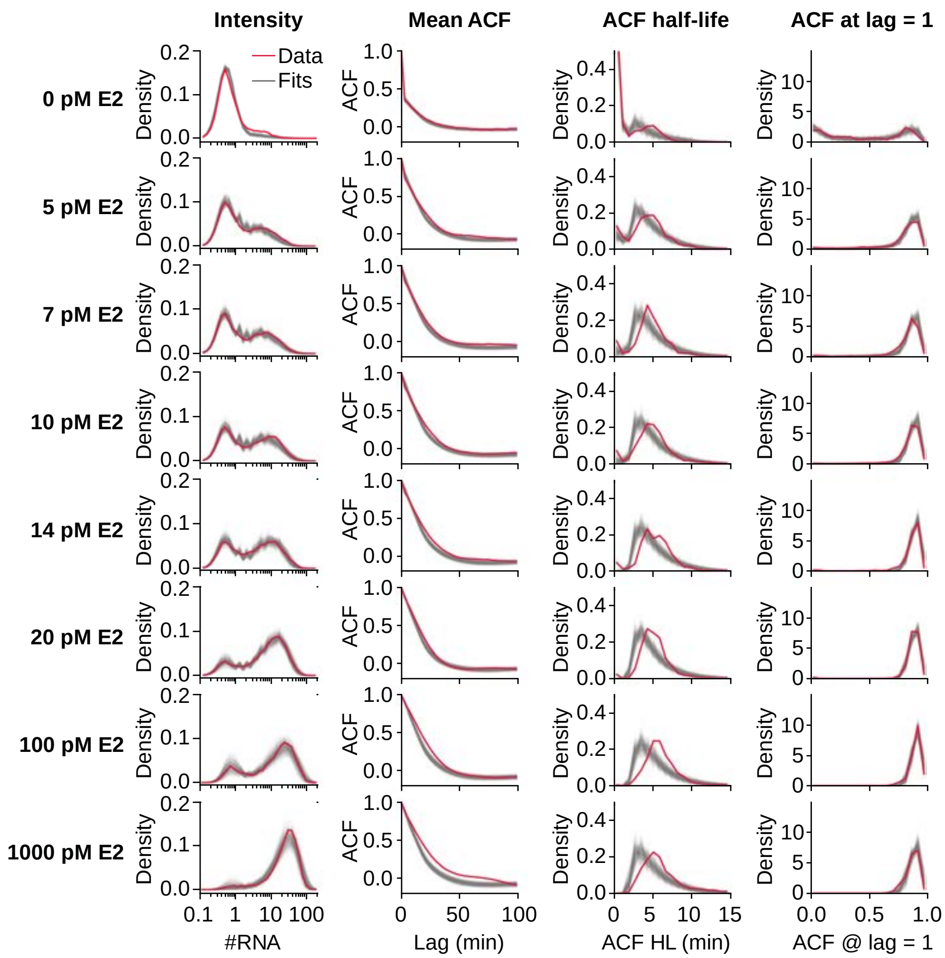

Appendix Figure S4. Model fitting recapitulates features of experimental data. Related to Fig 3.

The main features for the data (red) and the 500 best particles obtained after SMC ABC (grey) are shown as in Fig 3B for all E2 concentrations.

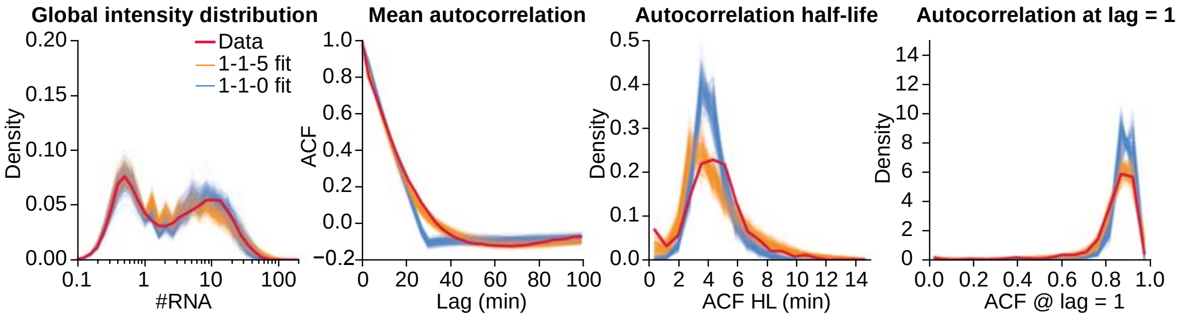

Appendix Figure S5. Features of fitted models with and without extrinsic noise sources. Related to Fig 3.

Model calibration was performed on the 10 pM E2 dataset with a fixed model topology. Either a two-state model without extrinsic noise (1-1-0, blue) or extrinsic variations in k_{elong} and k_{init} (1-1-5, orange) was fitted to the data. Plots show the features of the experimental data (red) alongside with features of the 400 best particles of each fit. The model with extrinsic variation in transcription achieves a better approximation of the autocorrelation function-based features.

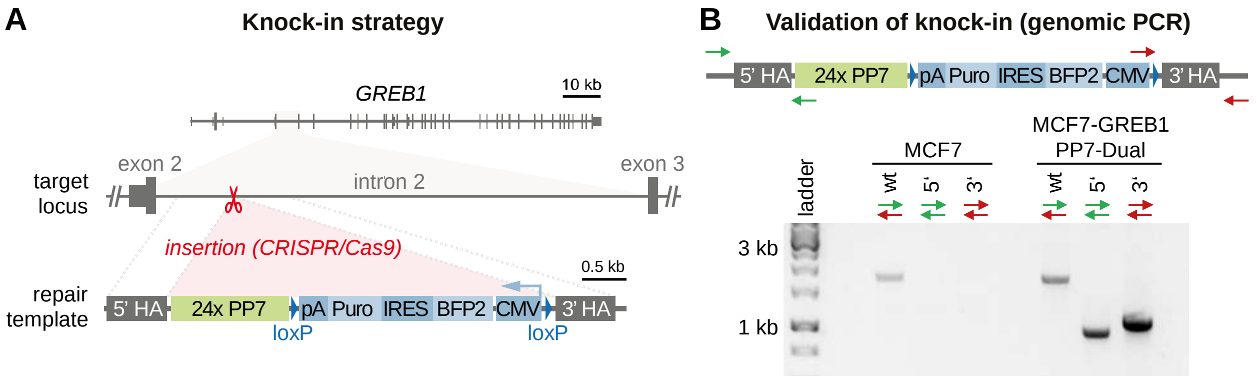

Appendix Figure S6. Genome engineering of the dual allele cell line. Related to Fig 5.

(A) *Knock-in strategy to integrate PP7 sequences into intron 2 of GREB1 in MCF-7 cells*. PP7 sequences were knocked-in together with a selection cassette into intron 2 of *GREB1* using CRISPR/Cas9 genome engineering. (HA: homology arm; pA: polyadenylation site; Puro: Puromycin resistance; IRES: internal ribosomal entry site; CMV: promoter of cytomegalovirus).

(B) *Validation of genome engineering.* Genotyping PCRs were performed on genomic DNA with primers that are positioned along the transgene as indicated (top). PCR products confirm successful knock-in after Cas9-mediated cleavage on both 3' and 5' ends of the construct (bottom). At least one *GREB1* wild-type allele (wt) remains in the knock-in clone, as indicated by the PCR product for the wild-type locus. This suggests that more than two *GREB1* loci are present in the parental MCF-7 cell line.

Single-molecule RNA FISH (Dual allele cell-line)

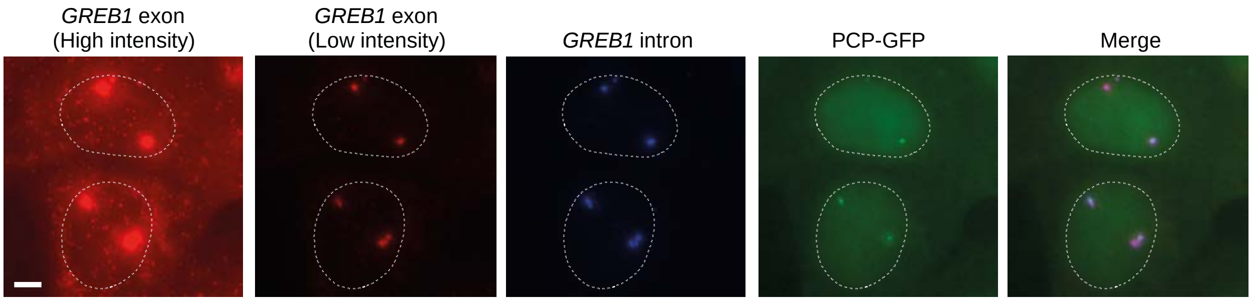

Appendix Figure S7. Single-molecule RNA FISH of GREB1 overlaps with GFP signal at two out of three transcription sites in the dual allele cell line. Related to Fig 5.

smRNA FISH images of the dual allele cell line with exonic (red) and intronic (blue) *GREB1* probes show an overlap with PCP-GFP spots (green) at two out of three nuclear spots. Scale bar: 5 µm. Cells were stimulated with 100 pM E2.

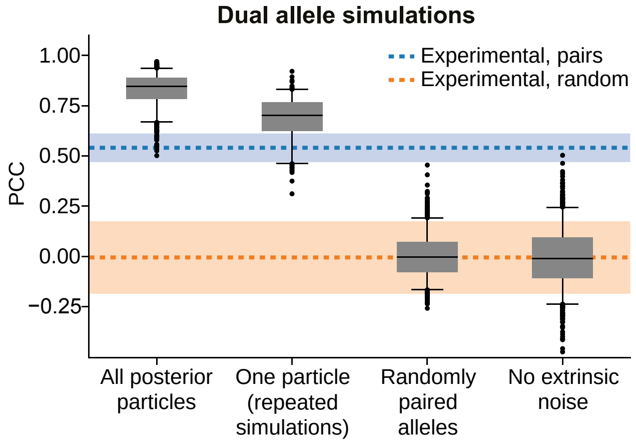

Appendix Figure S8. Simulated sister allele correlations agree with experimental measurements in the dual allele cell line. Related to Fig 5.

Sister alleles were simulated by assuming the same extrinsic noise realization (k_{init} and k_{elong}) in two independent simulation runs. The Pearson correlation coefficient (PCC) was calculated to quantify the relation of the total RNA output of 45 sister alleles. The left boxplot shows the spread of PCCs for all posterior two-state particles (1-1-5 model) at 10 pM E2 in Fig 3, one sister allele simulation being performed per particle. “Randomly paired alleles” and “no extrinsic noise” reflect the corresponding PCCs of randomly paired trajectories and of sister allele trajectories in the absence of extrinsic fluctuations, respectively. The column “One particle” represents the PCC spread of one particular two-state posterior particle for which 200 (independent) simulations were performed to assess statistical variation arising from limited sample sizes. Horizontal lines and shading represent mean ± standard deviation from bootstrapping of the experimentally observed PCC between sister alleles (blue) or randomly reassigned pairs (orange).

Boxplots: central line: median, box: 25 % and 75 % percentile, whiskers: 5 % and 95 % percentile.

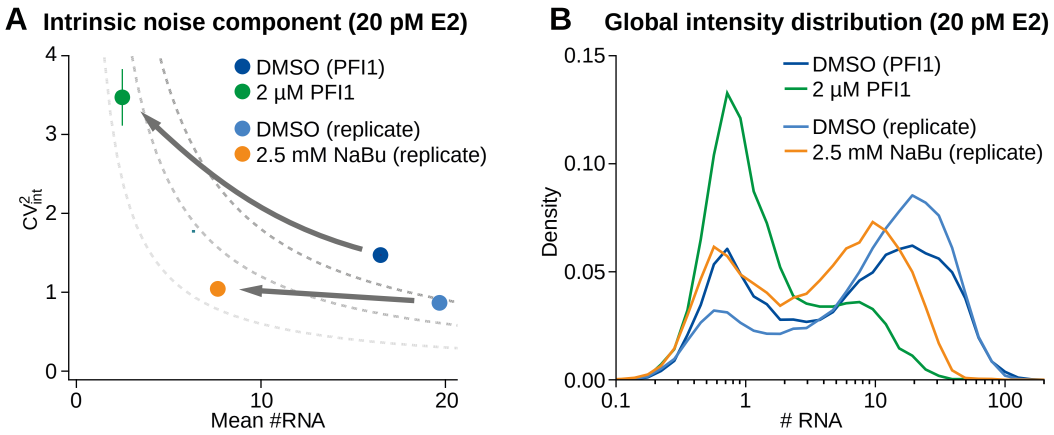

Appendix Figure S9. Intrinsic noise component and global histogram for PFI1 treatment and replicate of NaBu treatment. Related to Fig 7.

(A) Cells were grown at 20 pM E2 and treated with DMSO, PFI1, or sodium butyrate (NaBu) for 4 hours prior to imaging. Intrinsic noise (CV^2) is plotted against mean expression, with butyrate lowering mean *GREB1* expression levels without affecting noise as compared to solvent control. PFI1 also lowers expression levels and only slightly increases noise, comparable to 4 mM butyrate treatment (Fig 7D). Dashed lines indicate inverse noise-mean relation for different burst sizes according to an analytical model of intrinsic noise, in which mean expression is controlled by changing burst frequencies (CV^2 ~ burst size/$mean_{RNA}$).

(B) Global intensity histograms for experiments in panel A.

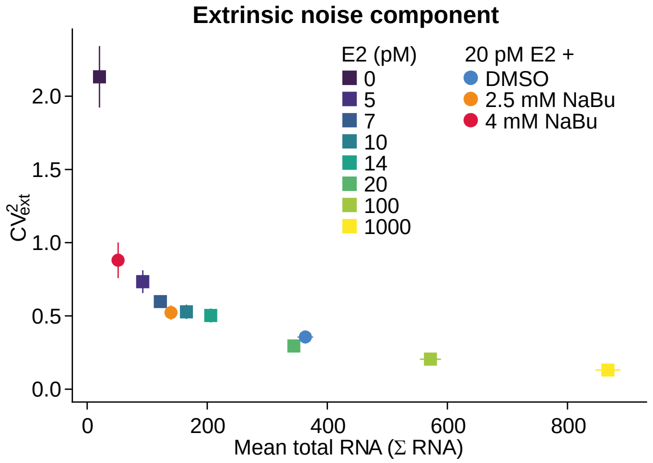

Appendix Figure S10. Extrinsic noise component for E2 dose-response and HDACi treatment. Related to Fig 7.

Extrinsic noise is estimated by calculating the squared coefficient of variation over the total RNA outputs of all cells. HDAC inhibition (red and orange dots) does not alter extrinsic noise when compared to the extrinsic shifts induced by changing estrogen concentrations.

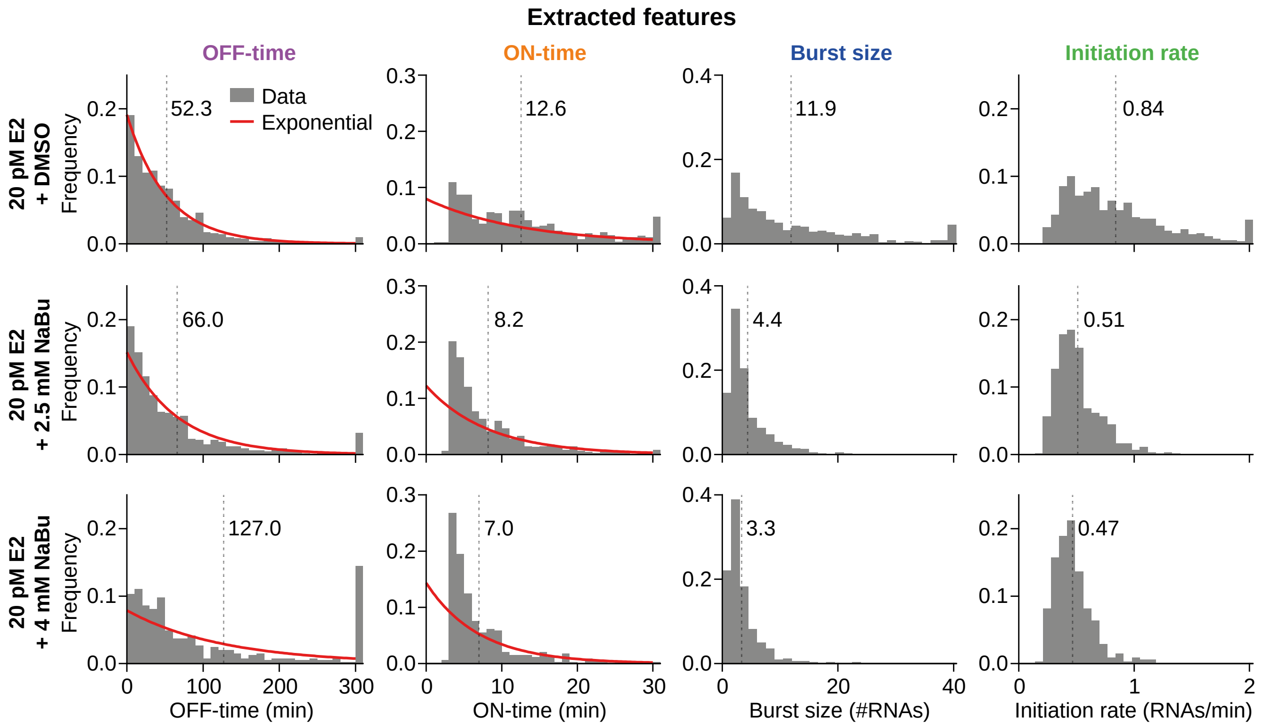

Appendix Figure S11. Features extracted from single-cell time courses confirm burst size modulation by butyrate. Related to Fig 7.

Features were extracted as in Fig EV2E-F. An exponential distribution with the same mean (dashed line) as the feature is shown in red. 2.5 mM butyrate leads to a reduction in burst size (ON-time*initiation rate) while a further increase in butyrate concentration (4 mM) additionally affects the burst frequency (OFF-time).

Appendix Tables

Appendix Table S1: Oligonucleotides for cloning. Related to Fig 1, 5, and EV4.

Name	Sequence
gRNA_ex2_t	CACCGTTTCACCTTCTACCTTGCG
gRNA_ex2_b	AAACCGCAAGGTAGAAGGTGAAAC
gRNA_int2_t	CACCGTCTCACACAAGCTCAGTGTG
gRNA_int2_b	AAACCACACTGAGCTTGTGTGAGAC
inF_PCPlinker_GPF_for	GCTGTACAAGTCCGGACTCAGATCTCGAGCTCAAGCTTCGAATTCTGTGAGCAAGGGCGAGGAGCT
inF_GFP_rev	TTATCTAGATCCGGTGGATCCCGGGTTACTTGTACAGCTCGTCCATGCCG
inF_K2L_PCP_for	ACCGTCAGATCCGCTAGTGCCCGCCATGGGCCCAAA
inF_GFP_PCP_rev	CTGAGTCCGGACTTGTACAGCTCGTCCATGCCG
tdPCP_for	ATTTAGCGGCCGCCATGTCCAAAACCATCGTTCTTTCGGTC
tdGFPSV40_rev	GCCTCATCGATGTTAAGATACATTGATGAGTTTGGACAAACC
pSB_for	AGGCAATCGATCCCAAGTTAAACAATTTAAAGGCAATGC
pSB_rev	AACCTACTAGTCCAAGCTGTTTAAAGGCACAGTCAA
inF_ex2_L_pUC_for	CGACGGCCAGTGAATTCAGTGGTTCTCTGTGATTTTTGGG
inF_ex2_L_loxP_rev	TACCAACTAGTGATCAAGGTGAAACAGCTGCAAGGA
inF_ex2_R_loxP_for	TCTGATAGTTGCTTATGCACCGAATCTGAGATGCCA
inF_ex2_R_pUC_rev	TCGACTCTAGAGGATCACTGAGGGGTTACTTCTGA
inF_int2_L_PP7_rev	ATTCGTTTAAACCTGCAGGAGATCTCACACAAGCTCAGTGTGGAGCCCCTTGAAGACAA
inF_int2_L_pUC_for	AGTCGACCTGCAGGCATGCATCAGTAGGAGAAAGGAAGAGGAC
inF_int2_R_PP7_for	CTCGCGAAGGGCGAATTCGCTGATAGTTGCTTATTCTAGCAGGATGCATTTCTGT
inF_int2_R_pUC_rev	CTATGACCATGATTACGCCAAGGTGGGCAGAAAGCAACTA
inF_loxP_R_for	TTATCTGATAGTTGCTTATTCTAGCAGGATGC
inF_loxP_L_rev	ATAACTTCGTATAATGTATGCTATACGAAGTTATAAGCGGCCGCTACCAACTAGTGATCTCACACA AGCTCAGTGTGG
inF_BFP_IRESPuro_for	AAGTCCAAGCTGTAATGCATCTAGGGCGGCCAA
inF_loxP_Puro_rev	ATTATACGAAGTTATCCATAGAGCCCACCGCATC
inF_loxP_CMVBFP_for	GCAACTATCAGATAACTTCGTATAATGTATGCTATACGAAGTTATAACCGTATTACCGCCATG
inF_IRES_BFP_rev	TTACAGCTTGGACTTGTACAGCTCGTC
inF_BFP_Puro_for	GATCACTAGTTGGTAGCGGCC
inF_BFP_Puro_rev	TAAGCAACTATCAGATAACTTCGTATAATGTATG
inF_GAPDH_pUC_for	AAAACGACGGCCAGTGAATTTGCCTCCTGCACCACCAAC
inF_GAPDH_GREB1wt_rev	AAGGTGAAACCCACAGTCTTCTGGGTGGCAG
inF_GREB1wt_GAPDH_for	AAGACTGTGGGTTTCACCTTCTACCTTGCGTGGA
inF_GREB1wt_ki_rev	AGAGACGAAGGCAAGACCTCTTCAAAGCGTGTC
inF_GREB1ki_wt_for	GAGGTCTTGCCTTCGTCTCTGCTGAGCGAAGG
inF_GREB1ki_pUC_rev	CAGGTCGACTCTAGAGGATCTTAGGTACCTTAGGATCCCGCGAAG

Appendix Table S2: Oligonucleotides for genotyping PCRs. Related to Fig EV1 and EV5. The amplicon size is given for different cell lines: wt: MCF-7, ex: MCF7-GREB1-PP7-BFP-Puro, dual: MCF7-GREB1-PP7-Dual, rec: MCF7-GREB1-PP7. Parenthesis denote that the amplicon is too big to be amplified in the chosen PCR conditions.

<table>
<tr><th>Name</th><th>Sequence</th><th colspan="3">Amplicon size (bp)</th></tr>
<tr><td>PP7loxP_for</td><td>TAGATCTCGCGAAGGGCGAA</td><td></td><td rowspan="2"></td><td rowspan="4">ex: (5018); rec: 946</td></tr>
<tr><td>GREB1_int2_3’_rev2</td><td>CCCCCTTGTTTTCCTGCACTAGA</td><td rowspan="2">dual: 1080</td></tr>
<tr><td>HR_CMV_for</td><td>ACCGTAAGTTATGTAACGCGGAACT</td><td rowspan="2">ex: 1012</td></tr>
<tr><td>GREB1_ex2_3’_rev</td><td>TGCGGGTAACTTCAAGTCAAAG</td><td rowspan="2">wt: 1943; ex: (6483); rec: (3492)</td></tr>
<tr><td>GREB1_ex2_5’_for</td><td>CCATCCCTTCCCATCTGCAAG</td><td colspan="2" rowspan="2">ex: 1108; rec: 1108</td></tr>
<tr><td>HR_PP7_rev</td><td>TACCTTAGGATCCCGCGAAG</td><td rowspan="2">dual: 947</td></tr>
<tr><td>GREB1_int2_5’_for</td><td>CTAGAAGGTGGGAGACGCAC</td><td colspan="2" rowspan="2">wt: 1745; dual: (6292)</td></tr>
<tr><td>GREB1_int2_3’_rev</td><td>CATACCAACGTGGAGCTGGA</td><td></td></tr>
</table>

Appendix Table S3: Oligonucleotide pairs for RT-qPCRs. Related to Fig EV1 and EV4.

Name	Sequence	Target	Amplicon size (bp)
GREB1_ex2wt_for<br>GREB1_ex2_rev	ACCTTCTACCTTGCGTGGAG<br>CCTCTTCAAAGCGTGTCGTC	*GREB1*, wt allele	129
GREB1_ex2_for<br>HR_PP7_rev	TCTCTGCTGAGCGAAGGC<br>TACCTTAGGATCCCGCGAAG	*GREB1*, PP7 allele	107
GREB1_exint2_for<br>GREB1_exint2_rev	GTCCAACAACCTGGTGCC<br>CAGATAAAAGCAACGTGCGTC	*GREB1,* exon2-intron2	104
GREB1_intex33_for<br>GREB1_intex33_rev	GCCGCTTTCCTCTGGATAAAC<br>GATGACACACAACGTCGCA	*GREB1,* intron32-exon33	82
GAPDH_for<br>GAPDH_rev	CTGCACCACCAACTGCTTAG<br>GTCTTCTGGGTGGCAGTGAT	*GAPDH*	108

Appendix Table S4: Probes for single-molecule RNA FISH of intronic regions of *GREB1*. Related to Fig EV1 and Appendix Fig S7.

Probes were ordered from LGC Biosearch Technologies labeled with Quasar® 670.

#	Sequence	Target
1	AGAAGTCTGCGGGTAACTTC	*GREB1* intron 2
2	CACAGTCTAGTTTCTCTCAG	*GREB1* intron 2
3	AAGCTGAGTATGCAACTGCT	*GREB1* intron 2
4	TTTTTATCTCAGAGCTTGGC	*GREB1* intron 2
5	TGTCCAACAGCAGATAAGGG	*GREB1* intron 2
6	AGTGCTTGGTTTAGGGTAAC	*GREB1* intron 2
7	CCAGGTTTGACTATGCAGAA	*GREB1* intron 2
8	CCCGACAAACAGACACTACG	*GREB1* intron 2
9	GATCAAGGGGTGTTCAGTAT	*GREB1* intron 2
10	AACAAGCTCGAGGGGACATT	*GREB1* intron 2
11	CAGACTCTCAGAAGGCATGA	*GREB1* intron 2
12	AATAAGGGAGACACATCCCA	*GREB1* intron 2
13	CACAGATGCCAACTATCAGT	*GREB1* intron 2
14	CCATGTCGGACACAGAAGAC	*GREB1* intron 2
15	CGTGGTTTTAGCAGAAGGTG	*GREB1* intron 2
16	GGCTAAGCAACCTAGGTTAA	*GREB1* intron 2
17	AGGATCTCAGGCTGTTGAAA	*GREB1* intron 2
18	GGTACACAGAATCGTACCTT	*GREB1* intron 2
19	ATTATGACTGTGTGTTGGGC	*GREB1* intron 2
20	ATGAAATTCTCTCAGCTCCG	*GREB1* intron 2
21	AACAGGGTCAGGTATGGTAT	*GREB1* intron 2
22	GTAGATGGTACCATAGTGTC	*GREB1* intron 2
23	TAACAAGCACTCAGCACGTC	*GREB1* intron 2
24	TAATCCAGAATGGGTTCCAC	*GREB1* intron 2
25	TTTAGGTTTGTCTCAGGAGG	*GREB1* intron 9
26	CTGATCAGGGGCTGAGCTAG	*GREB1* intron 9
27	GACAAGCTCCATCATTCTTG	*GREB1* intron 9
28	CATGGCAGGGAATCATTTCA	*GREB1* intron 9
29	CGTGGGTGATAACAGGAGAC	*GREB1* intron 9
30	TTGATGCTTCTACAGTGGTG	*GREB1* intron 9
31	TCAGCTGAGCCAAAAGTCTA	*GREB1* intron 9
32	AGGGTTTCCCATTGCAACAT	*GREB1* intron 9
33	ATTCTGGTGCCTACATATCA	*GREB1* intron 9
34	AACGTATTTAAGAGGGGCCT	*GREB1* intron 9
35	TTAACAGTAGGGTGCTTCTC	*GREB1* intron 9
36	GCGTCATGCTAAGGTCGAAA	*GREB1* intron 9
37	TGGAACTGCCTCGGTCATTG	*GREB1* intron 9
38	TTTCTTTTCCGAGGTTCCTG	*GREB1* intron 9
39	TGTGAGACGTAGACTTGCTG	*GREB1* intron 9
40	AGCAGAGCACGCCTGAGAAC	*GREB1* intron 9
41	GAGTCTTAAGGCCTCAGGAG	*GREB1* intron 9
42	CACAGTGACTTCATGACGTC	*GREB1* intron 10
43	TCAACCTCAACCTACTTTCA	*GREB1* intron 10
44	TTCATGACCTAAACTGACCC	*GREB1* intron 10
45	GGGACTATGAGAAAGAGCGA	*GREB1* intron 10
46	CGTGCTTACTGATGGACAGG	*GREB1* intron 10
47	AATCGGAGTCCAAGTTCTCA	*GREB1* intron 10
48	ACTATTCCCTCAATATAGGT	*GREB1* intron 10

Appendix Table S5: Models and parameter values to create synthetic benchmarking data sets. Related to Fig EV3.

**Benchmark data set**	**Initiation rate (#RNA/min)**	**ON-time (min)**	**Burst size (# RNA)**	**OFF-time (min)**
1-1 0	7	10	70	70
1-1 1	7	10	70	70
1-1 2	7	10	70	70
1-1 3	7	10	70	70
1-1 4	7	10	70	70
1-1 6	7	10	70	70
1-1 7	7	10	70	70
1-2 1	7	10	70	70
1-2 5	7	10	70	70
2-2 5	7	10	70	70
1-9 5	7	10	70	70
1-1 5 20 30	3.5	20	70	30
1-1 5 10 150	7	10	70	150
1-1 5 10 300	7	10	70	30
1-1 5 5 700	7	5	35	700
1-1 5 2 10	7	2	14	10
1-1 5 2 15	7	2	14	15
1-1 5 2 20	7	2	14	20
1-1 5 2 30	7	2	14	30
1-1 5 2 40	8	2	16	40
1-1 5 4 40	4	4	16	40
1-1 5 8 40	2	8	16	40

Appendix Movies

Movie EV1. *GREB1* is transcribed in infrequent and stochastic bursts, that are temporally resolvable at low induction. Related to Fig 1.

MCF7-GREB1-PP7 cells were grown in 5 pM E2. Images were acquired in the GFP channel every 3 minutes and are shown as cropped maximum intensity projections of z-stacks. Transcription sites are visible as single foci with fluctuating intensities within nuclei. Intensity values were clipped below 100 and above 450. The scale bar represents 10 µm.

Movie EV2. Active transcription of *GREB1* is observed for almost all cells at all times for saturating concentration of E2. Related to Fig 2.

MCF7-GREB1-PP7 cells were imaged at 1000 pM E2 every 3 minutes and cropped maximum intensity projections of z-stacks are shown. Transcription sites are apparent as bright foci within the nuclei with fluctuating intensities. Pauses in between bursts are rarely observed. The cell to the right of the image center initially shows two adjacent transcription sites, indicating a replicated allele in G2 phase of the cell cycle. This cell undergoes mitosis and both daughter cells continue transcription after cytokinesis. Intensity values are clipped below 100 and above 350. The scale bar represents 10 µm.

Movie EV3. Single cells show heterogeneous timing in their transcriptional response after induction with E2. Related to Fig 4.

After starving MCF7-GREB1-PP7 cells from E2, they were imaged every 1.5 minutes for five hours. 1000 pM E2 was added after 51 minutes and transcriptional bursting starts after different response times in individual cells. Cropped images are shown as maximum intensity projections of z-stacks and the time before and after addition of 1000 pM E2 is indicated. Intensity values are clipped below 95 and above 300. The scale bar represents 10 µm.

Movie EV4. Bursts of individual alleles within the same cell are not correlated. Related to Fig 5.

MCF7-GREB1-PP7-Dual cells were imaged at 10 pM E2 every 3 minutes for 12.5 hours. Two distinct transcription sites are visible in each cell and their intensity fluctuates independently. Cropped maximum intensity projections of z-stacks are shown with intensity values being clipped below 100 and above 300. The scale bar represents 10 µm.

Appendix Datasets

Dataset EV1-6. Raw fluorescence trajectories for all live-cell imaging experiments in this study.

Appendix References

Ai, H., Shaner, N.C., Cheng, Z., Tsien, R.Y., and Campbell, R.E. (2007). Exploration of new chromophore structures leads to the identification of improved blue fluorescent proteins. Biochemistry *46*, 5904–5910.

Angers, S., Thorpe, C.J., Biechele, T.L., Goldenberg, S.J., Zheng, N., MacCoss, M.J., and Moon, R.T. (2006). The KLHL12-Cullin-3 ubiquitin ligase negatively regulates the Wnt-beta-catenin pathway by targeting Dishevelled for degradation. Nat. Cell Biol. *8*, 348–357.

Asch, V.B. Van (2012). Domain Similarity Measures: On the use of distance metrics in natural language processing. Universiteit Antwerpen.

Beaumont, M.A., Zhang, W., and Balding, D.J. (2002). Approximate Bayesian computation in population genetics. Genetics *162*, 2025–2035.

Cong, L., Ran, F.A., Cox, D., Lin, S., Barretto, R., Habib, N., Hsu, P.D., Wu, X., Jiang, W., Marraffini, L.A., Zhang, F. (2013). Multiplex genome engineering using CRISPR/Cas systems. Science *339*, 819–823.

Gillespie, D.T. (1977). Exact stochastic simulation of coupled chemical reactions. J. Phys. Chem. *81*, 2340–2361.

Gretton, A., Borgwardt, K.M., Rasch, M.J., Schoelkopf, B., and Smola, A. (2012). A Kernel Two-Sample Test. J. Mach. Learn. Res. *13*, 723–773.

Hunter, J., Dale, D., and Droettboom, M. (2007). Matplotlib.

Jaqaman, K., Loerke, D., Mettlen, M., Kuwata, H., Grinstein, S., Schmid, S.L., and Danuser, G. (2008). Robust single-particle tracking in live-cell time-lapse sequences. Nat. Methods *5*, 695–702.

Jones, E., Oliphant, T., Peterson, P., and Al, E. (2001). SciPy: Open source scientific tools for Python.

Larson, D.R., Zenklusen, D., Wu, B., Chao, J.A., and Singer, R.H. (2011). Real-time observation of transcription initiation and elongation on an endogenous yeast gene. Science *332*, 475–478.

Mátés, L., Chuah, M.K.L., Belay, E., Jerchow, B., Manoj, N., Acosta-Sanchez, A., Grzela, D.P., Schmitt, A., Becker, K., Matrai, J., Ma, L., Samara-Kuko, E., Gysemans, C., Pryputniewicz, D., Miskey, C., Fletcher, B., VandenDriessche T., Ivics, Z., Izsvák, Z. (2009). Molecular evolution of a novel hyperactive Sleeping Beauty transposase enables robust stable gene transfer in vertebrates. Nat. Genet. *41*, 753–761.

Del Moral, P., Doucet, A., and Jasra, A. (2006). Sequential Monte Carlo samplers. J. R. Stat. Soc. Ser. B Stat. Methodol. *68*, 411–436.

Otsu, N. (1979). A threshold selection method from gray-level histograms. IEEE Trans. Syst. Man. Cybern. *9*, 62–66.

Perez, F., and Granger, B.E. (2007). IPython : A System for Interactive Scientific Computing. IEEE 21–29.

Van Rossum, G., and Drake, F.L. (2003). The python reference manual (PythonLabs).

Sisson, S.A., Fan, Y., and Tanaka, M.M. (2007). Sequential Monte Carlo without likelihoods. Proc. Natl. Acad. Sci. U. S. A. *104*, 1760–1765.

Stoeger, T., Battich, N., Herrmann, M.D., Yakimovich, Y., and Pelkmans, L. (2015). Computer vision for image-based transcriptomics. Methods *85*, 44–53.

Tavaré, S., Balding, D.J., Griffiths, R.C., and Donnelly, P. (1997). Inferring coalescence times from DNA sequence data. Genetics *145*, 505–518.

Toni, T., Welch, D., Strelkowa, N., Ipsen, A., and Stumpf, M.P.H. (2009). Approximate Bayesian computation scheme for parameter inference and model selection in dynamical systems. J. R. Soc. Interface *6*, 187–202.

van der Walt, S., Colbert, S.C., and Varoquaux, G. (2011). The NumPy Array: A Structure for Efficient Numerical Computation. Comput. Sci. Eng. *13*, 22–30.

Woodhead, G.J., Mutch, C.A., Olson, E.C., and Chenn, A. (2006). Cell-autonomous beta-catenin signaling regulates cortical precursor proliferation. J. Neurosci. *26*, 12620–12630.

Wu, B., Chao, J. a, and Singer, R.H. (2012). Fluorescence Fluctuation Spectroscopy Enables Quantitative Imaging of Single mRNAs in Living Cells. Biophys. J. *102*, 2936–2944.

Zoller, B., Nicolas, D., Molina, N., and Naef, F. (2015). Structure of silent transcription intervals and noise characteristics of mammalian genes. Mol. Syst. Biol. *11*, 823.
