# Supplementary material for: Estrogen‐dependent control and cell‐to‐cell variability of transcriptional bursting
Source: Mol Syst Biol. 2018 Feb 23;14(2):e7678. doi: 10.15252/msb.20177678 (PMC5825209; doi:10.15252/msb.20177678)
Supplement: Supplementary file 2 — Expanded View Figures PDF [file MSB-14-e7678-s002.pdf]

Expanded View Figures

Figure EV1. Validation of knock-in cell line and image calibration (related to Fig 1).

A Validation of successful genome engineering. Genotyping PCRs were performed on genomic DNA with primers positioned along the transgene as indicated in the scheme above. PCR products confirm successful recombination after Cas9-mediated cleavage on both 3′ and 5′ ends of the construct, as well as successful Cre-mediated recombination between the two loxP sites (wt: wild-type locus, rec: locus after Cre recombination).

B The estrogen sensitivity of knock-in and wild-type (wt) allele is comparable. E2 dose-response curves were measured by allele-specific RT–qPCR after starving cells of E2 for 3 days followed by induction for 18 h at the indicated E2 concentrations (data points). Hill functions were fitted (lines), and the resulting EC_{50} is indicated. They confirm unperturbed sensitivity (EC_{50}) for E2 of the knock-in allele with minor differences in maximal RNA levels that most probably result from altered transcript stability. Error bars represent standard deviation from four biological replicates.

C The fraction of cells with visible transcription sites increases with E2 concentration. Transcription sites were automatically identified and quantified in fixed cells as in Fig 1D. The percentage of nuclei with detectable spots was obtained. Mean and standard deviation from three biological replicates are plotted alongside with the fit of a Hill function. ICI 182,780 (ICI) and actinomycin D (ActD) were applied at 100 pM E2.

D The single-molecule RNA FISH signal of exonic and intronic signal overlaps with the GFP signal at transcription sites. RNA FISH was performed with probes against exonic (red) and intronic (blue) regions of *GREB1*. Single RNAs are visualized by exonic probes as diffraction limited spots in the nucleus (dashed line) and cytoplasm of the knock-in cell line. Bright foci in the nucleus correspond to nascent RNA at the transcription sites that are visualized by intronic and exonic probes. One of three nuclear foci (arrowhead and inset) co-localizes with GFP signal from the PP7 system. Scale bars: 5 and 0.5 μm in the inset.

E Quantification of FISH signals determines a correlation between exonic, intronic, and GFP signal at transcription sites; 87% of bright nuclear exonic foci co-localize (distance < 5 px) with intronic foci, while only 26% of them co-localize with GFP foci, indicating that one in three foci is labeled with GFP. Co-localizing foci also correlate in intensity.

F Calibration of spot intensities by matching live-cell imaging distributions to absolute smRNA FISH signals. The mean intensity of single RNAs (solid vertical line) was derived from FISH images of *GREB1* exons at 100 pM E2. The intensity distribution of bright nuclear foci in smRNA FISH ($>$ 10 RNAs, red) was matched with the intensity distribution of transcription sites from live-cell imaging at 100 pM E2 (green), indicating that an estimated maximum of 150 RNAs occurs within a transcription site.

G Dose dependence of E2-dependent transcription. Nuclear smRNA FISH signals were quantified at various E2 concentrations. The mean intensity of the two brightest nuclear foci without GFP signal (wild-type alleles) is comparable to the mean intensity of the brightest spot co-localizing with GFP (knock-in allele). Both show an E2-dependent increase that is reduced upon addition of 1 μM ICI 182,780 (ICI).

H Absolute calibration of spot intensities by counting single PP7-labeled *GREB1* RNA molecules. (Left) MCF7-GREB1-PP7 cells were grown in 1,000 pM E2, and images were acquired at maximum light intensity. Single RNAs (red arrowheads) are apparent as dim spots, often in close proximity to the transcription site (blue asterisk). A maximum intensity projection of bandpass-filtered images is shown in the middle. The filtering accentuates particles which are then quantified by fitting to a three-dimensional Gaussian distribution, as seen on the right for the particle marked with the red arrowhead with white border. Scale bars: 5 and 1 μm for the single spot. (Middle) Intensity distribution of single transcripts. The histogram of spot intensities was fitted to a Gaussian function. The mean of the Gaussian function is indicated in the legend. (Right) Transcription sites were imaged at the same stage position under imaging conditions for long-term live-cell imaging (2% light intensity) and conditions for visualization of single transcripts (100% light intensity). Their intensities were quantified and the fitted ratio of intensities used to calculate the equivalent intensity of a single transcript under live-cell imaging conditions.

 © 2018 The Authors

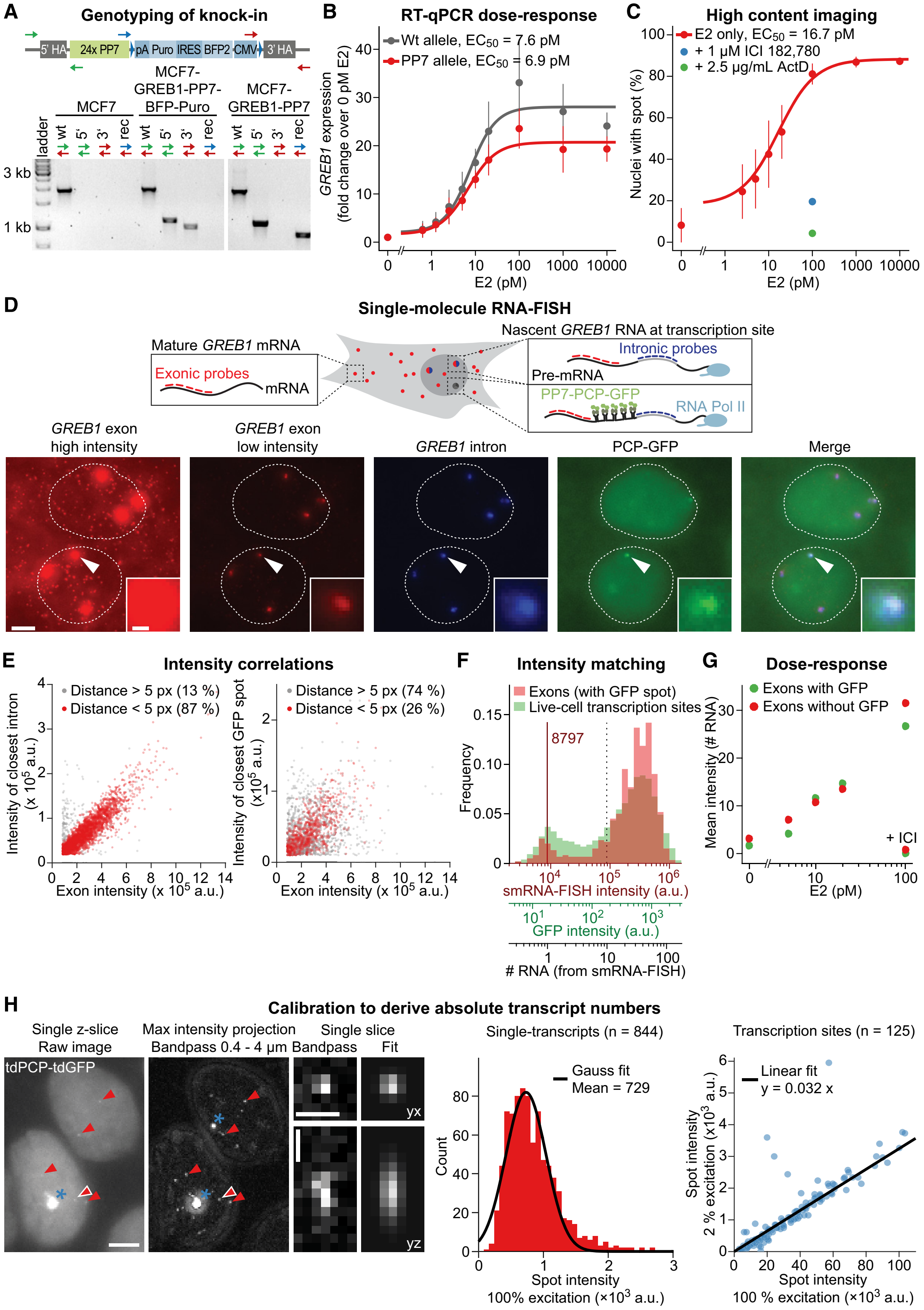
A
Genotyping of knock-in
5' HA
24x PP7
pA Puro IRES BFP2 CMV
3' HA
MCF7
MCF7-GREB1-PP7-BFP-Puro
MCF7-GREB1-PP7
ladder
wt
5'
3'
rec
3 kb
1 kb
B
RT-qPCR dose-response
Wt allele, EC50 = 7.6 pM
PP7 allele, EC50 = 6.9 pM
GREB1 expression (fold change over 0 pM E2)
E2 (pM)
C
High content imaging
E2 only, EC50 = 16.7 pM
+ 1 μM ICI 182,780
+ 2.5 μg/mL ActD
Nuclei with spot (%)
E2 (pM)
D
Single-molecule RNA-FISH
Mature GREB1 mRNA
Exonic probes
mRNA
Nascent GREB1 RNA at transcription site
Intronic probes
Pre-mRNA
PP7-PCP-GFP
RNA Pol II
GREB1 exon high intensity
GREB1 exon low intensity
GREB1 intron
PCP-GFP
Merge
E
Intensity correlations
Distance > 5 px (13 %)
Distance < 5 px (87 %)
Intensity of closest intron (x 10^5 a.u.)
Exon intensity (x 10^5 a.u.)
Distance > 5 px (74 %)
Distance < 5 px (26 %)
Intensity of closest GFP spot (x10^5 a.u.)
F
Intensity matching
Exons (with GFP spot)
Live-cell transcription sites
Frequency
8797
smRNA-FISH intensity (a.u.)
GFP intensity (a.u.)
RNA (from smRNA-FISH)
G
Dose-response
Exons with GFP
Exons without GFP
Mean intensity (# RNA)
+ ICI
E2 (pM)
H
Calibration to derive absolute transcript numbers
Single z-slice Raw image
tdPCP-tdGFP
Max intensity projection Bandpass 0.4 - 4 μm
Single slice Bandpass
Fit
yx
yz
Single-transcripts (n = 844)
Gauss fit
Mean = 729
Count
Spot intensity 100% excitation (×10^3 a.u.)
Transcription sites (n = 125)
Linear fit
y = 0.032 x
Spot intensity 2 % excitation (×10^3 a.u.)
Spot intensity 100 % excitation (×10^3 a.u.)

Figure EV1.

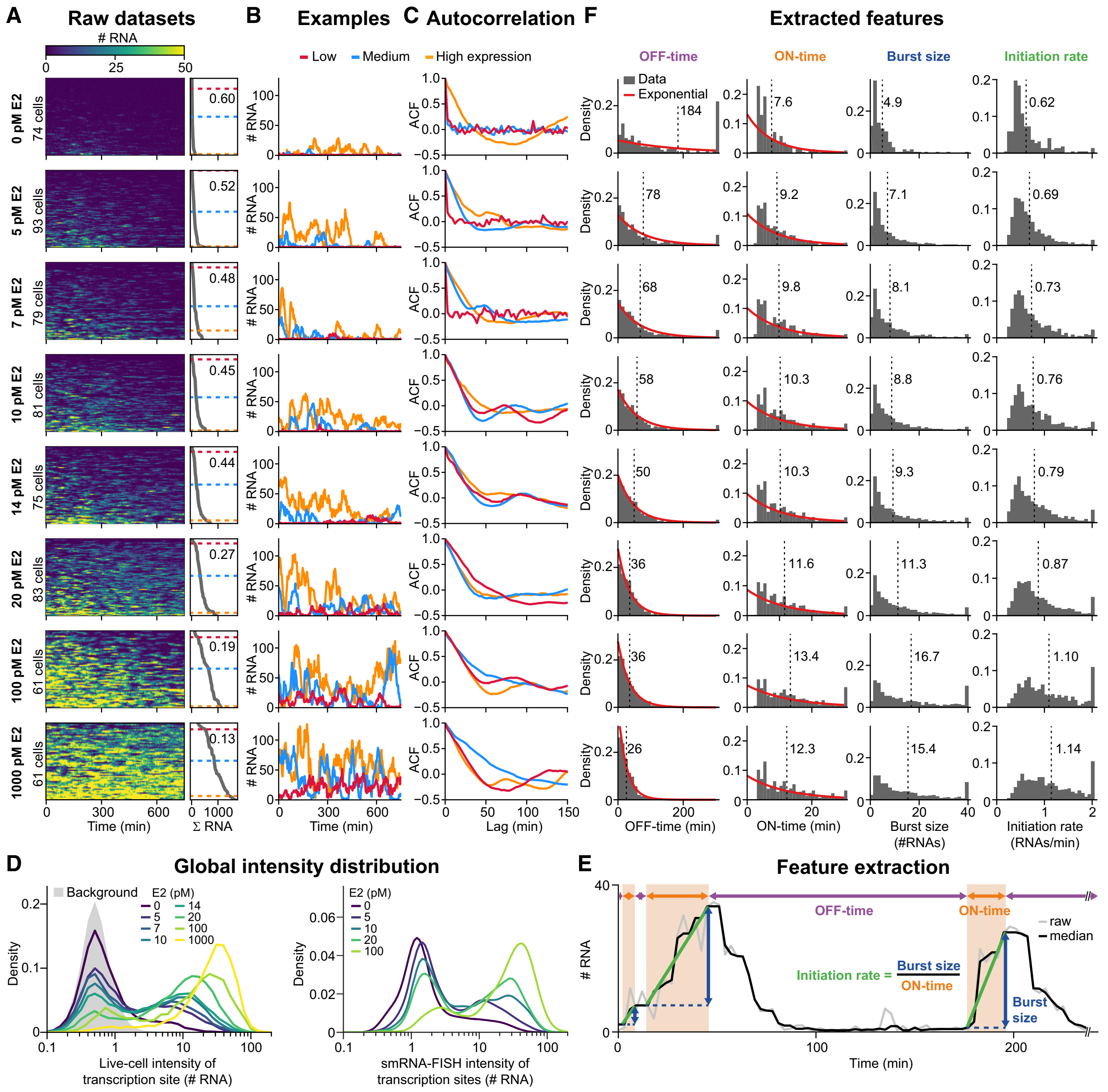

Figure EV2. *GREB1* transcription changes with estrogen concentration and exhibits considerable cell-to-cell variation throughout all datasets. Related to Fig 2.

A–C Raw data, example trajectories and autocorrelation functions (ACF) as in Fig 2, are shown for all eight E2 concentrations.

D Histograms of the number of RNA molecules at transcription sites. The distribution of nascent RNAs from all eight live-cell datasets over all cells and time points is plotted (left), with the background signal shown in gray. The intensity distribution from smRNA FISH (right) is comparable across E2 concentrations.

E Extraction of ON- and OFF-times from raw time traces. A simple thresholding strategy was used on the slope of the median-filtered time trace to separate transcriptionally active from inactive periods. The duration of each ON- and OFF-time was calculated. For each ON-time, the burst size was calculated as the increase in the number of nascent RNAs during an ON-time, and the initiation rate was derived as the ratio of burst size and ON-time.

F Distribution of OFF- and ON-times indicates that single rate-limiting steps occur during the transition between promoter states. Burst features were extracted from time traces in panel (A) as described above. The mean value is indicated in the plots (dashed line). Exponential distributions with the same mean are plotted for the OFF- and ON-times (red) and indicate good agreement. Durations in the range of the imaging interval cannot be reliably estimated leading to deviation at short ON-durations.

© 2018 The Authors

Figure EV3. Benchmarking of SMC ABC algorithm and fitting of experimental data. Related to Fig 3.

A Model fitting yields close-to-optimal description of synthetic data. Distance distributions of the final particle populations after SMC ABC fitting to synthetic data are shown in gray for each of the benchmark models (*y*-axis), box plots indicating the variance of the 2,000 best particles. For comparison and to estimate the best possible distance, the distance measure was calculated between 500 randomly paired simulations using the true model parameters based on which the synthetic data were generated (orange). The dashed gray line denotes a value of 0.5, which was chosen as the final convergence limit of the SMC ABC algorithm.

B SMC ABC accurately identifies true parameter values from benchmark datasets. Posterior distributions of model parameters for all benchmark datasets are presented. Red dots indicate the values used to generate the benchmarking datasets. Boxplots show posterior distributions for initiation rate, promoter ON-time, burst size, and promoter OFF-time.

C SMC ABC determines the correct model topology. The frequency of models in the posterior distribution is shown as color maps for all benchmark datasets in (A). Red dots mark the true model.

D Distances of the final particle populations after SMC ABC model fitting to datasets at various estrogen concentrations (Fig EV2A). The dashed gray line indicates a distance value of 0.5 for an optimal fit, as estimated in panel (A).

E Posterior parameter distributions. Boxplots show posterior distributions of burst size, ON-time, and OFF-time after SMC ABC fitting to all datasets.

F Model selection. The frequency of all forty models in all eight final particle populations after SMC ABC model fitting is shown. Only three models (*) were found in all eight posterior distributions (i.e., at all estrogen concentrations). The columns "Topology" and "Variability" indicate the number of ON/OFF promoter states and possible combinations of parameter resampling as specified on the *y*-axis in panel (C).

G Incorporating extrinsic noise in the model is necessary to fit the data at all E2 concentrations. The final distance distribution of particles after SMC ABC when fitting defined model topologies with and without extrinsic noise over different E2 concentrations is shown. Fitting was performed while fixing a two-state model topology that either includes no extrinsic noise (1–1–0) or a combination of extrinsic noise in k_{elong} and k_{init} (1–1–5). In addition, two models comprising a single source of extrinsic noise in either k_{elong} (1–1–1) or k_{init} (1–1–2) were fitted to the 10 pM dataset, with this combination of both sources yielding the best fit.

Data information: (A, B, D, E, G) Description of boxplots: central line, median; box, 25 and 75% percentile; whiskers, 5 and 95 percentile.

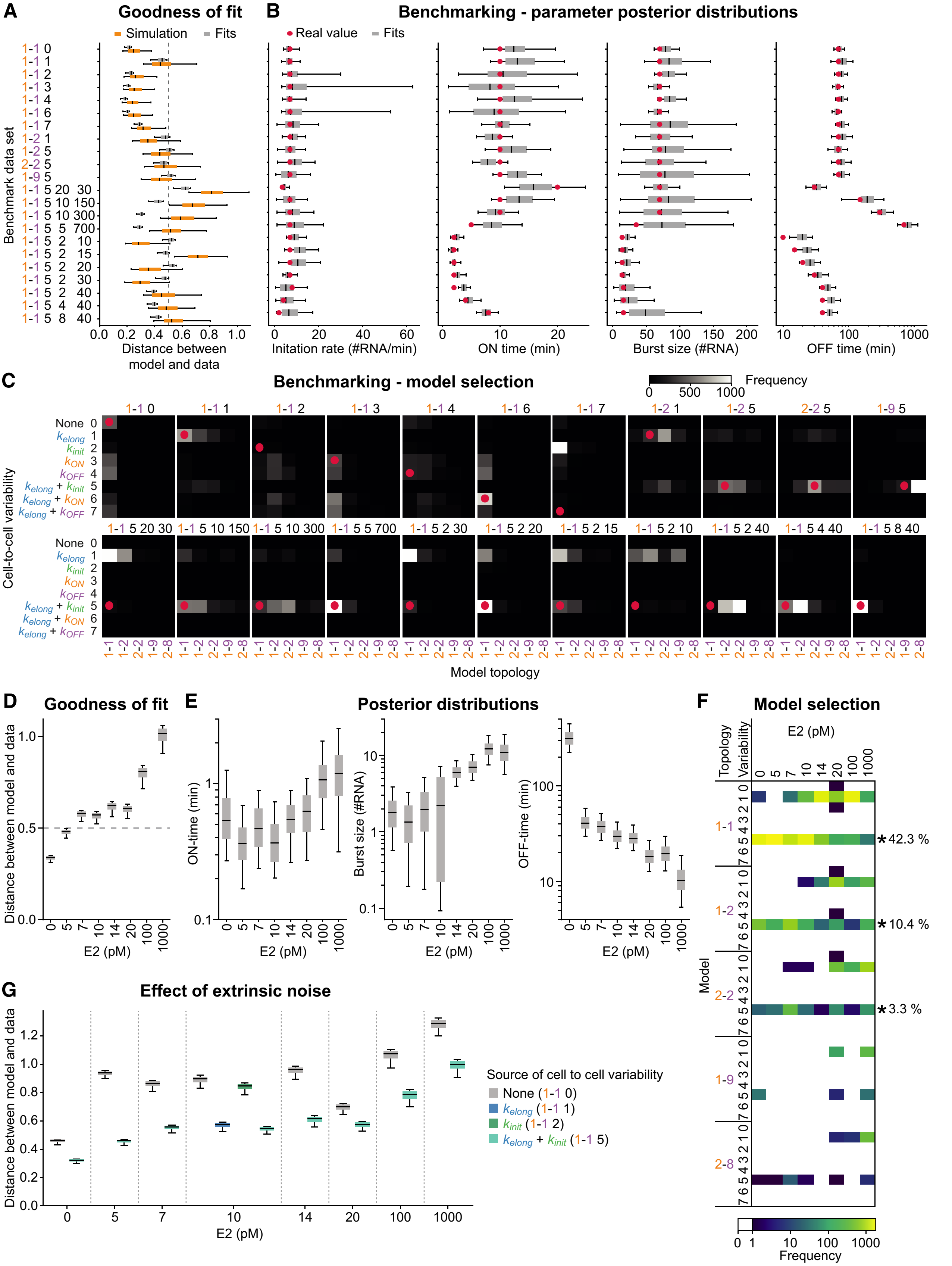

Figure EV3.

© 2018 The Authors

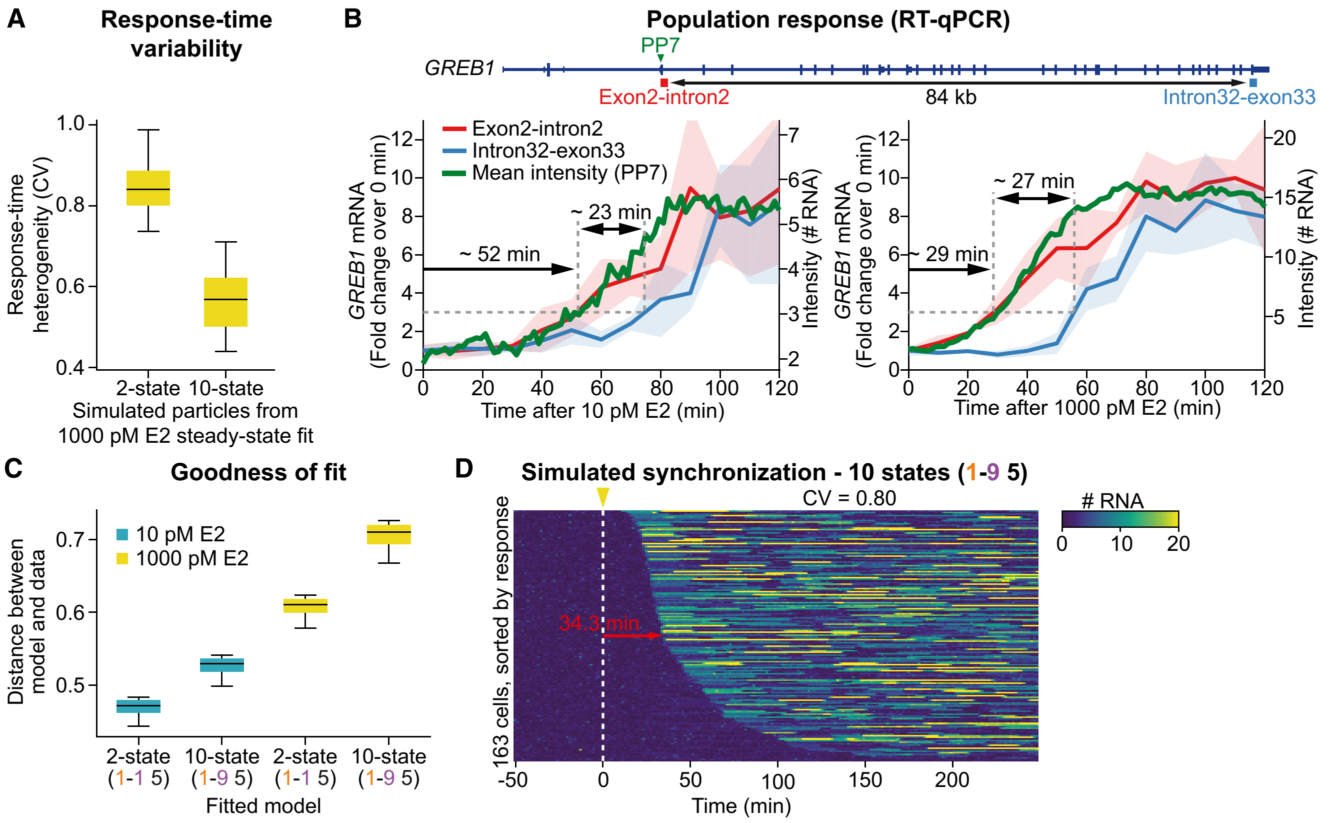

Figure EV4. Population kinetics and simulation of E2 induction. Related to Fig 4.

A Variability of response times depends on the number of steps in the promoter cycle. Heterogeneity in response times was calculated as the coefficient of variation (CV) from simulations of E2 induction experiments. The distribution of CVs over all SMC ABC posterior particles from the fit to steady-state dose-response at 1,000 pM E2 (Fig 3) with models containing either two (left) or ten (right) states is shown as boxplots.

B The kinetics of E2 induction in single living cells is similar to ensemble RT–qPCR measurements. MCF7 cells were starved of E2 for 3 days and then induced with either 10 pM (left) or with 1,000 pM E2 (right) with samples being collected every 10 min for RT–qPCR. The location of the qPCR products and of the PP7 sequences is indicated on the schematic of the *GREB1* gene structure. Primer pairs were designed to span exon–intron boundaries to exclusively amplify unspliced pre-mRNA. The mean of three experiments is plotted with standard deviation shown as shaded areas. Response times and delay between amplicons are estimated from the time taken to achieve a threefold induction. The mean intensity of transcription sites from Fig 4A is shown in green and shows excellent agreement with population and single-cell response.

C Minimal promoter models yield a better model fit for induction datasets. SMC ABC was performed with the 10 or 1,000 pM E2 induction datasets, while keeping the model topology fixed at a two-state (1–1–5) or ten-state (1–9–5) model. Distances of the final particle populations are shown as boxplots. For both experimental datasets, the two-state model yields a smaller final distance value than the ten-state model, indicating better description of the data.

D Promoter models with multiple states produce homogenous response times. Simulations of synchronized cells were performed with parameters derived from fits of the 10 pM induction dataset for a promoter cycle with 10 states (t_{ON} = 0.8 min; t_{OFF} = 30 min; b = 6 RNAs/burst, model topology: 1–9–5). The median and CV of response times are indicated in red.

Data information: (A, C) Description of boxplots: central line, median; box, 25 and 75% percentile; whiskers, 5 and 95% percentile.

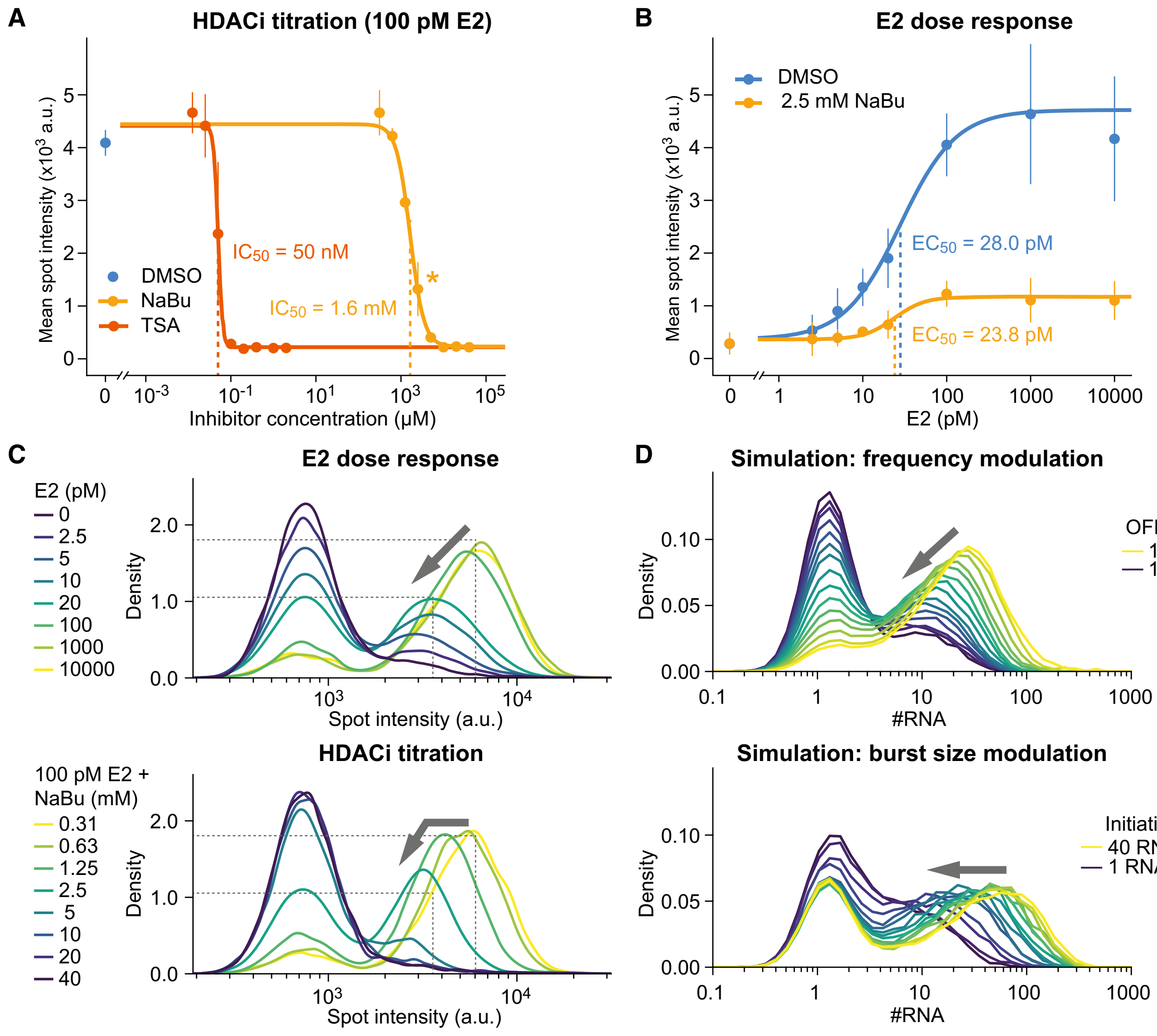

Figure EV5. Effect of HDAC inhibition on the steady-state and dynamic transcriptional response and associated noise. Related to Fig 7.

A HDAC inhibitors have a dose-dependent effect on transcriptional activity. MCF7-GREB1-PP7 cells were cultured with 100 pM E2 for 3 days, and then, butyrate (NaBu) or TSA was added for 4 h prior to fixation. Mean transcription sites intensities of > 5,000 cells per condition were calculated and fitted to a Hill function. Error bars denote standard deviation from two replicates. An intermediate butyrate concentration (*) was chosen for E2 titration experiments in panel (B).

B Inhibitors of deacetylation alter the intensity of transcription sites. MCF7-GREB1-PP7 cells were cultured at various concentrations of E2 for 3 days, after which 2.5 mM butyrate (NaBu) was added for 4 h. Transcription sites were detected automatically and quantified using > 2,500 cells per condition. The mean and standard deviation from two biological replicates are plotted and fitted to a Hill function.

C Intensity histograms of HDAC inhibitor titration reveal qualitative differences in noise behavior as compared to the E2 dose-response. Kernel density estimates were calculated from spot intensities from high-content imaging datasets (same data as in panel A and B). Comparison with stochastic simulations (panel D) indicates a decrease in burst size at low NaBu concentrations, as compared to the E2 dose-response. Low NaBu doses primarily affect intensity but not the frequency, of the right peak in the histogram (see arrow).

D Simulated intensity histograms distinguish burst size from burst frequency modulation. Stochastic simulations were performed (t_{ON} = 1 min; b = 8 RNAs/burst; model topology: 1–1–5) with OFF-times ranging from 10 (yellow) to 100 min (blue) and global intensity histograms were calculated. Both the proportion and intensity of active transcription sites changes with OFF-time (top). Burst sizes were changed by increasing the initiation rate from 1 (blue) to 40 RNAs/min (yellow) during stochastic simulations (t_{ON} = 1 min; t_{OFF} = 30 min; model topology: 1–1–5) (bottom). Only the intensity of active transcription sites (x-position of the right peak) changes with changing burst sizes.

 © 2018 The Authors
